# COPS5 Triggers Ferroptosis Defense by Stabilizing MK2 in Hepatocellular Carcinoma

**DOI:** 10.1002/advs.202416360

**Published:** 2025-04-08

**Authors:** Ai‐Ling Luo, Wen‐Ying Zheng, Qiong Zhang, Yan Yuan, Mei‐Qi Li, Kai Du, An‐Ran Gao, Li‐Jun Pei, Jie Xie, Wen‐Hao Chen, Long Zhang, Xiu‐Zhu Guo, Xiao‐Ran Yang, Chao Zeng, Guo‐Hua Yang, Min Deng

**Affiliations:** ^1^ Guangzhou Institute of Cancer Research the Affiliated Cancer Hospital Guangzhou Medical University Guangzhou 510095 China; ^2^ Department of Hematology and Oncology Guangzhou Women and Children's Medical Center Guangzhou Medical University Guangdong Provincial Clinical Research Center for Child Health Guangzhou 510623 China; ^3^ Department of Laboratory Medicine Shunde Hospital Guangzhou University of Chinese Medicine Foshan 528300 China; ^4^ Department of Pathology the Eighth Affiliated Hospital Sun Yat‐sen University Shenzhen 518033 China

**Keywords:** COPS5, ferroptosis, hepatocellular carcinoma, sorafenib, therapeutic resistance

## Abstract

Sorafenib, which is proven to serve as a potent ferroptosis inducer, is used as a first‐line treatment for patients with advanced hepatocellular carcinoma (HCC), but it has limited clinical benefits, mainly due to drug resistance. Herein, using genome‐wide CRISPR/Cas9 knockout screening and multiple functional studies, this work identifies COP9 signalosome subunit 5 (COPS5) as a driver of sorafenib resistance and a suppressor of ferroptosis in HCC. Consistently, the amplification and overexpression of COPS5 are frequently observed in clinical HCC samples, which are associated with poor patient prognosis and might predict patient response to sorafenib therapy. Mechanistically, COPS5 stabilized mitogen‐activated protein kinase 2 (MK2) through deubiquitination and, in turn, induced the activation of heat shock protein beta‐1 (HSPB1), a ferroptosis repressor, thereby protecting HCC cells from ferroptosis and consequently leading to sorafenib resistance and tumor progression, while its own expression could be induced by sorafenib treatment via activating transcription factor 4 (ATF4)‐activated transcription. Furthermore, pharmacological inhibition of COPS5/MK2 synergize with sorafenib to induce ferroptosis and suppress HCC progression. This data reveals the crucial role of COPS5 in triggering ferroptosis defense and sorafenib resistance through the activation of the MK2‐HSPB1 axis in HCC and highlights the potential of targeting COPS5/MK2 combined with sorafenib as a promising strategy for treating HCC.

## Graphical abstract

COPS5, which is overexpressed in HCC in an amplification‐ and ATF4‐dependent manner, stabilizes MK2 through deubiquitination and, in turn, induces HSPB1 activation, protecting HCC cells from ferroptosis and thus promoting sorafenib resistance and tumor progression.

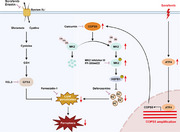

## 1 Introduction

Liver cancer is one of the most common malignancies and the third leading cause of cancer‐related mortality worldwide, with 75 7948 deaths (7.8%) in 2022.^[^ ^1^ ^]^ Hepatocellular carcinoma (HCC), which originates from hepatocytes, is the predominant subtype of primary liver cancer and accounts for ≈90% of all liver cancer cases. While surgical resection is recommended for patients with early‐stage HCC, those with advanced‐stage HCC often receive systemic therapies, including chemotherapeutics and tyrosine kinase inhibitors. Sorafenib, an oral multikinase inhibitor that mainly targets rapidly accelerated fibrosarcoma, c‐KIT, platelet‐derived growth factor receptor and vascular endothelial growth factor receptor‐1, is the first molecular‐targeted drug approved by the United States Food and Drug Administration (FDA) for first‐line treatment of advanced HCC, considerably improving clinical outcomes for a subset of patients.^[^ ^2^ ^]^ Another multikinase inhibitor, lenvatinib, was subsequently approved as a first‐line treatment for advanced HCC; however, it failed to provide a better overall survival than sorafenib.^[^ ^3^ ^]^ Although immunotherapy has opened up new strategies for HCC treatment, only a small subset of patients respond to immunotherapy. Sorafenib remains the standard therapy for advanced HCC, as supported by strong evidence and clinical experience. Unfortunately, the overall response rate to sorafenib is only ≈30%, which prolongs the median overall survival of patients with HCC by 2.8 months.^[^ ^2^, ^4^, ^5^ ^]^ Intrinsic or acquired drug resistance is a major obstacle causing the failure of sorafenib therapy in patients with HCC.^[^ ^6^ ^]^

The main mechanisms underlying sorafenib resistance in cancers include the activation of alternative signaling pathways, such as the phosphoinositide 3‐kinase‐protein kinase B (AKT), epidermal growth factor receptor, mammalian target of rapamycin and nuclear factor‐κB pathways, and the induction of hypoxia‐inducible factors (HIF)‐1α or HIF‐2α owing to tumor hypoxia linked to sorafenib therapy.^[^ ^6^ ^]^ However, the molecular mechanisms that drive sorafenib resistance in HCC have not been clearly elucidated, and no efficient therapies targeting specific resistance‐related mechanisms have been approved for patients with sorafenib‐resistant HCC. Therefore, it is critical to gain better insight into the molecular mechanisms of sorafenib resistance to target the root causes of drug resistance in HCC.

Ferroptosis, a form of programmed cell death driven by Fe^2+^‐dependent lipid peroxidation, is involved in various pathological processes, including acute tissue failure, neurodegenerative disorders, and cancer.^[^ ^7^ ^]^ Intriguingly, cancer cells are more susceptible to ferroptosis than normal cells are, likely because they use higher levels of iron and lipid metabolites to maintain aggressive growth.^[^ ^8^ ^]^ Therefore, ferroptosis induction has been proposed as a promising therapeutic strategy against cancer.^[^ ^9^ ^]^ Accumulating evidence has suggested that the antitumor effect of sorafenib is mainly due to the induction of ferroptosis through inhibition of the critical System Xc‐ and that insensitivity to ferroptosis is closely associated with sorafenib resistance.^[^ ^10^, ^11^ ^]^ Therefore, increasing the susceptibility of cancer cells to ferroptosis is expected to improve the efficacy of sorafenib.

In the present study, we performed CRISPR/Cas9 knockout (KO) screening for HCC using two mouse models to systematically identify the molecular drivers of sorafenib resistance. Based on these data, we prioritized a common hit COP9 signalosome subunit 5 (COPS5), which is the catalytic subunit of the COP9 signalosome complex that inactivates Cullin‐RING E3 ubiquitin ligases through deneddylation of the ubiquitin‐like activator NEDD8,^[^ ^12^ ^]^ for validation as a critical mediator of sorafenib resistance in HCC, and to determine its negative regulation of ferroptosis. Moreover, we demonstrated that COPS5 amplification and overexpression in HCC samples were associated with an inferior prognosis and poor response to sorafenib therapy. Further investigation has revealed that COPS5 interacts with and stabilizes mitogen‐activated protein kinase 2 (MK2) through deubiquitination, promoting the phosphorylation of heat shock protein beta‐1 (HSPB1), which is critical for ferroptosis suppression, rendering HCC cells resistant to sorafenib treatment. Sorafenib treatment upregulated COPS5 expression by activating transcription factor 4 (ATF4)‐activated transcription. We further demonstrated that pharmacological inhibition of COPS5/MK2 acts synergistically with sorafenib to induce ferroptosis and attenuate HCC progression, suggesting that targeting the COPS5‐MK2‐HSPB1 axis is a potential therapeutic strategy against therapy resistance in HCC.

## 2 Results

### 2.1 CRISPR/Cas9 KO Screening Identifies COPS5 as a Candidate Driver for Sorafenib Resistance in HCC

**Figure 1:**
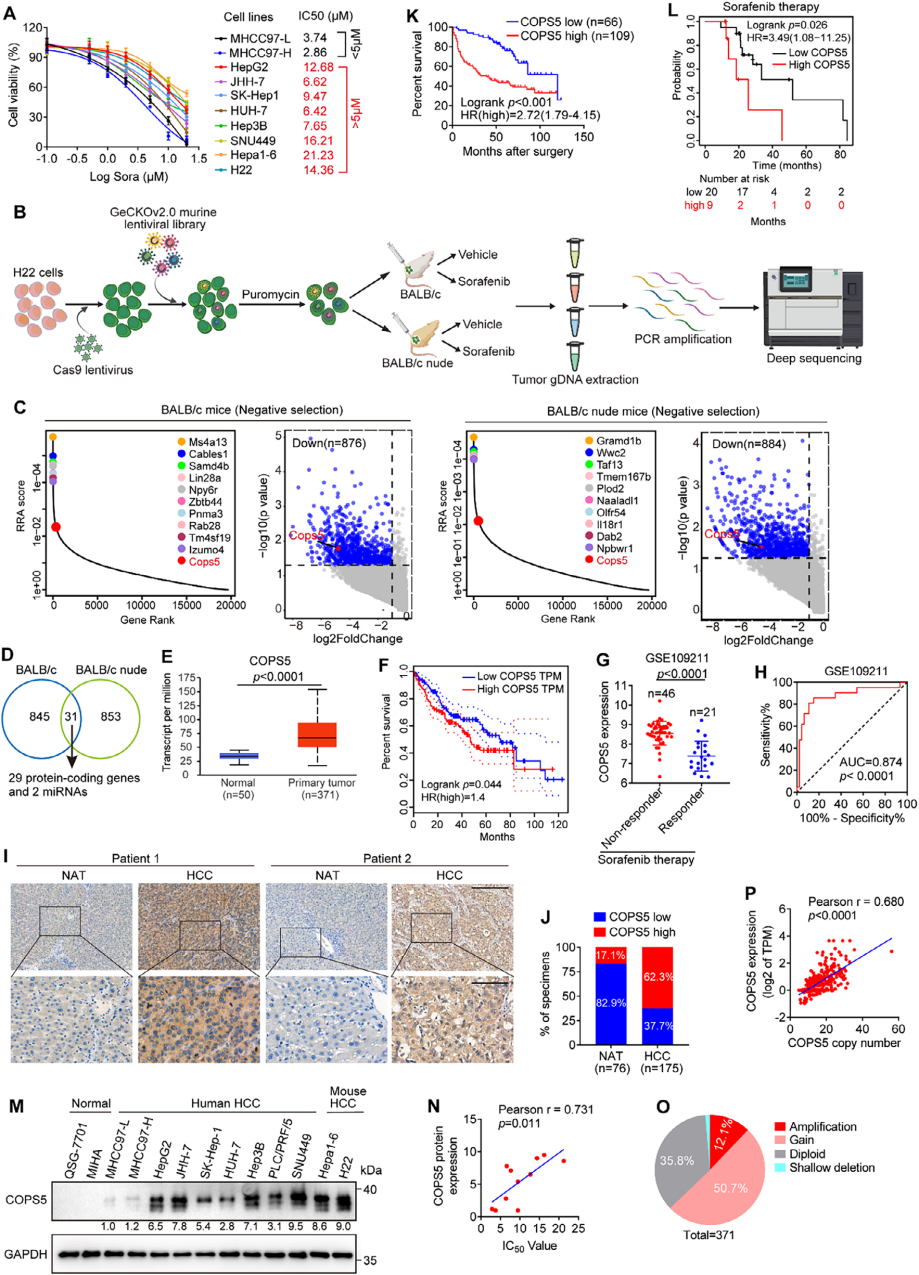
CRISPR/Cas9 knockout screening indicates COPS5 as a candidate driver for sorafenib resistance in HCC. A) The viability of HCC cells treated with various concentrations of sorafenib (Sora) for 72 h was tested using the CCK‐8 assay, and the IC50 values were determined. B) Schematic overview of the workflow of the CRISPR/Cas9 knockout screening. This figure was created via BioRende.com. C) Robust ranking aggregation (RRA) scores and volcano plots showing the genes depleted in sorafenib‐treated versus vehicle‐treated mice via CRISPR/Cas9 screening. D) Venn diagram showing the overlap of the depleted genes in the two sorafenib‐treated mouse models. E) COPS5 expression in HCC versus normal liver tissues from the TCGA–LIHC dataset was analyzed in the UALACN database. F) Association between COPS5 expression and overall survival was analyzed using TCGA–LIHC data via the GEPIA website. G) COPS5 expression in HCC tissues from sorafenib non‐responders and responders in the GEO dataset GSE109211.^[^ ^13^ ^]^ H) ROC curve depicting the correlation between COPS5 expression and responsiveness to sorafenib in patients with HCC from the GSE109211 dataset.^[^ ^13^ ^]^ I, J) IHC analysis of COPS5 expression in 175 HCCs and 76 NATs. Scale bar: 300 (upper) and 100 µm (lower). K) Overall survival curves for patients with HCC based on COPS5 expression as determined by IHC staining. L) Association between overall survival and COPS5 expression in patients with HCC receiving sorafenib therapy according to the Kaplan‐Meier plotter database. M) The protein levels of COPS5 in HCC and normal hepatic cell lines. N) Correlations between COPS5 protein levels and the sorafenib IC50 in HCC cell lines. O) Copy number alterations of COPS5 in HCC samples from cBioPortal using the TCGA dataset. P) Correlation between COPS5 expression and copy number in TCGA–LIHC samples. Data are presented as mean ± SD. Statistical analysis was conducted using a two‐tailed *t*‐test (E and G), a log‐rank test (F, K, and L), and a two‐tailed Pearson's test (N and P).

We first investigated the activity of sorafenib in 10 HCC cell lines using a CCK‐8 viability assay and demonstrated that, consistent with the limited clinical responses, most HCC cell lines are intrinsically resistant to sorafenib (**Figure** **1**A). To systematically identify the critical regulators involved in sorafenib resistance, we conducted in vivo CRISPR/Cas9 KO screening in the murine HCC cell line H22 using a GeCKOv2.0 murine lentiviral library containing 13 0209 sgRNAs targeting 20 611 protein‐coding and 1175 miRNA mouse genes. First, H22 cells were engineered to express Cas9 and then infected with the GeCKOv2.0 murine lentiviral library. After puromycin selection, the library‐transduced cells were subcutaneously implanted into syngeneic BALB/c mice and immune incompetent BALB/c nude mice, followed by sorafenib or vehicle treatment for 14 days. Allograft tumors were subjected to next‐generation sequencing (Figure 1B). Through negative screening, we identified 29 protein‐coding genes whose sgRNAs were depleted following sorafenib treatment in both mouse models (Figure 1C,D and Table S1, Supporting Information), implying that the depletion of these genes may have a synergistic antitumor effect with sorafenib. An examination of the clinical relevance of these genes in human HCC using the The Cancer Genome Atlas (TCGA) liver hepatocellular carcinoma (LIHC) dataset revealed significant upregulation of 15 genes in HCC samples compared with normal liver tissues, of which COPS5, ZRANB2, TSPAN17, HAUS1, KDELR3, and CDK5R1 levels demonstrated a notable positive correlation with worse overall survival (OS) in patients with HCC (Figure 1E,F; Figure S1A,B, Supporting Information). Next, we examined whether the expression levels of these genes were correlated with responsiveness to sorafenib in a cohort of 67 patients with HCC who received sorafenib therapy via the Gene Expression Omnibus (GEO) dataset GSE109211.^[^ ^13^ ^]^ The results revealed that patients with HCC who were non‐responsive to sorafenib treatment had higher levels of COPS5, ZRANB2, or TSPAN17 than those who responded well to sorafenib (Figure 1G; Figure S1C, Supporting Information). COPS5, which has deubiquitination activity attributed to the inactivation of cullin‐RING E3 ligases through deneddylation,^[^ ^14^ ^]^ has been implicated in tumor progression and therapeutic resistance.^[^ ^15^, ^16^, ^17^ ^]^ Therefore, we focused on COPS5 in the subsequent studies.

In TCGA pan‐cancer analysis, we found that COPS5 expression levels were higher in tumor tissues than in normal tissues in most cancer types (Figure S2A, Supporting Information). In addition to HCC, elevated COPS5 levels were associated with poor OS in breast invasive carcinoma (BRCA), kidney chromophobe (KICH), lung adenocarcinoma (LUAD), and pancreatic adenocarcinoma (PAAD) (Figure S2B, Supporting Information). To investigate whether COPS5 expression levels can predict the response of patients with HCC to sorafenib therapy, receiver operating characteristic (ROC) analysis was conducted. The analysis results showed that the area under the curve (AUC) was 0.874, indicating that COPS5 could be used as a predictive biomarker for the response to sorafenib therapy (Figure 1H). Subsequent immunohistochemistry (IHC) analysis of 175 HCC samples and 76 normal adjacent tissues (NATs) confirmed that COPS5 expression was higher in HCC tissues than in NATs and that high COPS5 expression was associated with inferior OS in patients with HCC (Figure 1I–K). We further demonstrated that in patients with HCC who received sorafenib treatment, participants with high COPS5 expression had a shorter OS time than those with low COPS5 expression in the Kaplan–Meier plotter database (Figure 1L). Additionally, most HCC cell lines expressed high levels of COPS5, whereas COPS5 expression was almost undetectable in human hepatic cell lines (Figure 1M). More importantly, in a panel of HCC cell lines with varying degrees of COPS5 expression, there was a negative correlation between COPS5 expression and sorafenib sensitivity (Figure 1N), suggesting its potential role in driving sorafenib resistance in HCC.

To further explore the mechanism underlying the upregulation of COPS5 in HCC, we conducted a genomic alteration analysis using TCGA–LIHC cohort in the cBioPortal database. As shown in Figure 1O, 12.1% (45/371) and 50.7% (188/371) of the HCC samples exhibited amplification and gain of the COPS5 locus, respectively. Moreover, COPS5 copy number was strongly associated with increased COPS5 mRNA expression (Figure 1P), suggesting that genomic amplification may be one of the mechanisms contributing to COPS5 overexpression in HCC. Kaplan–Meier survival analysis revealed that patients with COPS5 amplification had shorter OS times than those without COPS5 amplification (Figure S1D, Supporting Information). Collectively, these findings indicate that COPS5 amplification and overexpression may contribute to sorafenib resistance and disease progression in HCC.

### 2.2 COPS5 Depletion Sensitizes HCC Cells to Sorafenib and Suppresses HCC Cell Growth

**Figure 2:**
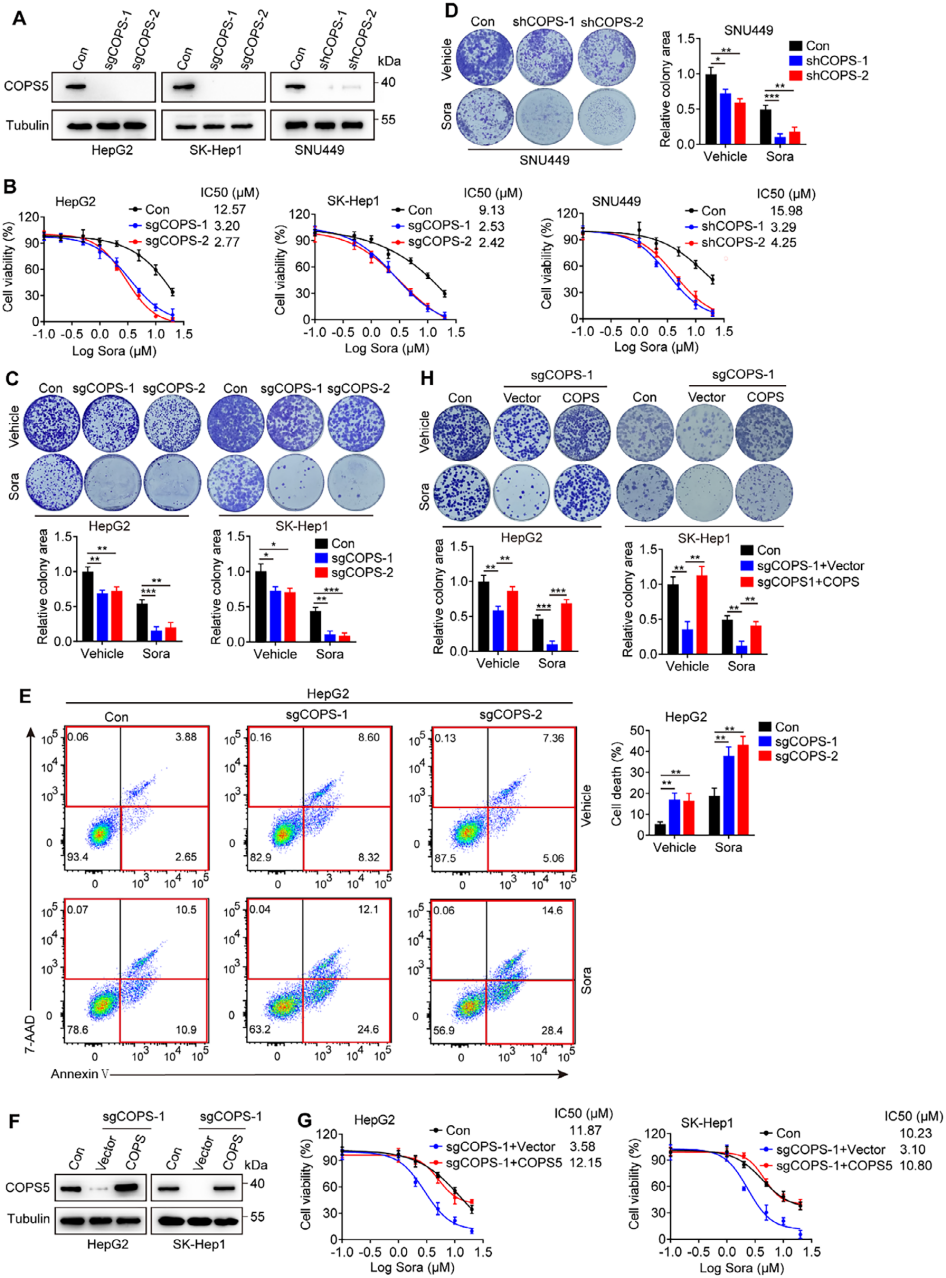
COPS5 depletion sensitizes HCC cells to sorafenib in vitro. A) Western blots showing COPS5 expression in COPS5‐KO/KD and control cells. B) Cell viability of COPS5‐KO/KD and control cells upon treatment with sorafenib for 72 h. C,D) Colony formation analysis of control, COPS5‐KO (C), and COPS5‐KD (D) cells treated with 10 µM sorafenib or vehicle. E) Cell death in COPS5‐KO and control cells treated with 10 µM sorafenib or vehicle for 48 h as quantified via Annexin V‐APC/7AA‐D staining. F) Western blots verifying COPS5 re‐expression in COPS5‐KO cells. G,H) Cell viability (G) and colony formation ability (H) of COPS5‐KO cells with or without COPS5 re‐expression and control cells following treatment with sorafenib or vehicle. Data are mean ± SD. *p < 0.05, ** *p* < 0.01, ***p < 0.001 (two‐tailed *t*‐test).

To confirm the results of our screening, we knocked out COPS5 using the CRISPR/Cas9 technique in the human HCC cell lines HepG2 and SK‐Hep1 as well as in the murine HCC cell line H22 (**Figure** **2**A; Figure S3A, Supporting Information) and subsequently examined their response to sorafenib. Cell viability and colony formation assays showed that all COPS5‐KO cell lines were more sensitive to sorafenib treatment, with three to fivefold lower IC50 values for sorafenib than the respective control cells (Figure 2B,C; Figure S3B,H, Supporting Information). Similar results were obtained by shRNA‐mediated COPS5 knockdown (KD) in the parental HCC cell line SNU449 and the sorafenib‐resistant cell subline HUH‐7R (Figure 2A,B,D; Figure S3C–E, Supporting Information), which were derived from the HCC cell line HUH‐7 by continuous selection with sorafenib. Flow cytometry revealed that COPS5 knockout enhanced sorafenib‐induced cell death in HepG2 and SK‐Hep1 cells (Figure 2E; Figure S3I, Supporting Information). Additionally, the viability of COPS5‐KO cells treated with sorafenib was rescued by the re‐expression of COPS5 (Figure 2F–H). COPS5 deficiency modestly inhibited cell growth and induced death in HCC cells without sorafenib treatment (Figure 2C–E; Figure S3H,I, Supporting Information), suggesting a role for COPS5 in HCC progression. Additionally, a stronger effect on sorafenib sensitivity in cells was observed upon KD of COPS5 than upon KD of MT1G, which has previously been shown to trigger sorafenib resistance in HCC (Figure S3D–G, Supporting Information).^[^ ^18^ ^]^ Taken together, these results indicate that ablation of COPS5 suppresses HCC cell growth and sensitizes HCC cells to sorafenib treatment.

### 2.3 COPS5 Protects HCC Cells from Ferroptotic Cell Death

**Figure 3:**
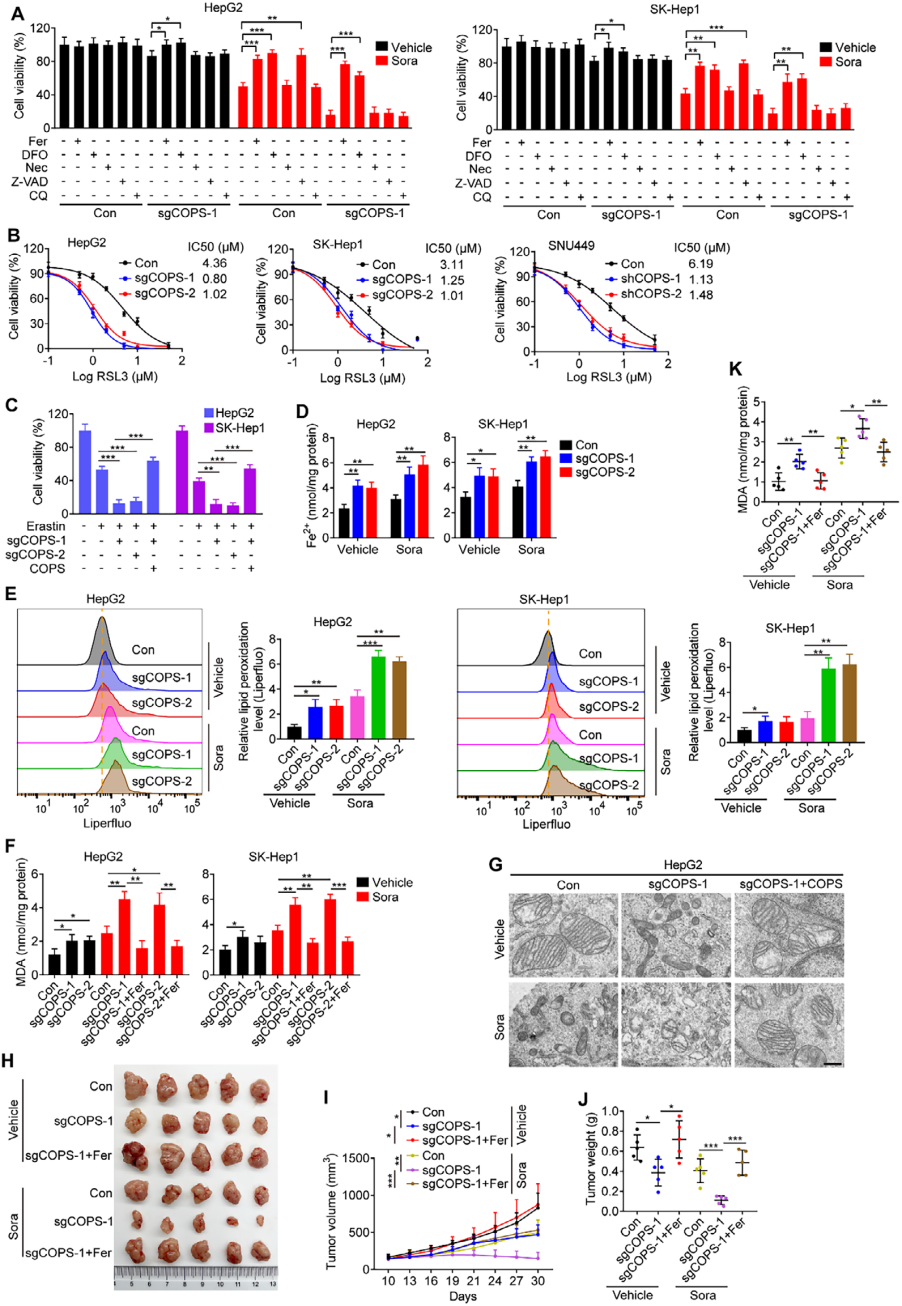
COPS5 acts as a ferroptosis repressor. A) The viability of COPS5‐KO and control cells was evaluated after combined treatment with sorafenib and a cell death inhibitor, 1 µM ferrostatin‐1 (Fer), 50 µM deferoxamine (DFO), 5 µM necrostatin‐1 (Nec), 10 µM Z‐VAD‐FMK (Z‐VAD), or 10 µM chloroquine (CQ) for 48 h. B) Viability of COPS5‐KO/KD and control cells after 48 h of incubation with increasing concentrations of RSL3. C) Cell viability in response to 5 µM erastin in control cells, COPS5‐KO cells, and COPS5‐KO cells rescued with COPS5 re‐expression. D–F) Fe^2+^ (D), lipid peroxidation (E), and MDA (F) levels of COPS5‐KO and control cells after treatment as indicated. G) Transmission electron microscopy images displaying morphological changes in the mitochondria of COPS5‐KO HepG2 cells with or without COPS5 re‐expression and control cells after treated as indicated. Scale bar: 500 nm. H–K) COPS5‐KO HepG2 and control cells were subcutaneously implanted into BALB/c nude mice, and the mice were treated with vehicle or sorafenib alone or in combination with ferrostatin‐1 for 3 weeks (5 mice/group). Photographs of tumors (H), tumor volumes (I), tumor weights (J), and MDA levels (K) in the xenograft tumors are shown. Data are mean ± SD. *p < 0.05, ** *p* < 0.01, ***p < 0.001 (two‐tailed *t*‐test).

Given the well‐recognized role of sorafenib as a potent ferroptosis inducer,^[^ ^19^ ^]^ we investigated whether COPS5‐induced sorafenib resistance in HCC was a result of ferroptosis insensitivity. To address this, we applied the corresponding inhibitors of various forms of cell death to specifically inhibit ferroptosis (ferrostatin‐1 and deferoxamine), apoptosis (Z‐VAD‐FMK), necroptosis (necrostatin‐1), or autophagy (chloroquine) and observed that ferrostatin‐1, deferoxamine, and Z‐VAD‐FMK, but not necrostatin‐1 and chloroquine, significantly rescued the viability of control cells following sorafenib treatment, whereas only ferrostatin‐1 and deferoxamine markedly enhanced the viability of COPS5‐KO cells under sorafenib treatment (**Figure** **3**A). This indicates that alterations in ferroptosis may be involved in COPS5‐mediated sorafenib resistance. COPS5 knockout reduced, but COPS5 overexpression enhanced the viability of HCC cells after exposure to other ferroptosis inducers, such as RSL3 and erastin (Figure 3B,C). As the crucial hallmarks of ferroptosis are ferrous iron (Fe^2+^) accumulation and lipid peroxidation,^[^ ^20^ ^]^ the intracellular Fe^2+^ content and lipid peroxidation levels were assayed. COPS5 knockout increased the levels of intracellular iron, lipid peroxidation, and malondialdehyde (MDA), a metabolite of lipid peroxidation, in HCC cells in the absence or presence of sorafenib, and these effects were reversed by ferrostatin‐1 treatment (Figure 3D–F). Next, we observed the mitochondrial morphology of HCC cells via transmission electron microscopy. COPS5‐KO cells showed morphological characteristics of ferroptosis, such as shrunken mitochondria, reduced or absent mitochondrial crests, and condensed mitochondrial membrane densities^[^ ^19^ ^]^ (Figure 3G; Figure S4C, Supporting Information). Conversely, COPS5 re‐expression in COPS5‐KO cells restored their resistance to ferroptosis (Figure 3C,G; Figure S4A–C, Supporting Information).

To determine whether COPS5 depletion increases the sensitivity to sorafenib‐induced ferroptosis in vivo, we used a subcutaneous xenograft model. Consistent with our cellular studies, COPS5 KO reduced tumor growth and potentiated tumor growth suppression conferred by sorafenib in BALB/c nude mice bearing HepG2 subcutaneous xenografts, as detected by both tumor volume and weight, which could be offset by ferrostatin‐1 treatment (Figure 3H–J). Additionally, elevated levels of the products of lipid peroxidation, MDA, and 4‐hydroxynonenal (4‐HNE) were detected in COPS5‐KO tumors compared with control tumors in the presence and absence of sorafenib, whereas these effects were mitigated by ferrostatin‐1 (Figure 3K; Figure S4D, Supporting Information). Collectively, these results demonstrate that the ablation of COPS5 increases the susceptibility of HCC cells to ferroptosis inducers.

### 2.4 COPS5 Activates the MK2‐HSPB1 Axis by Stabilizing the MK2 Protein

**Figure 4:**
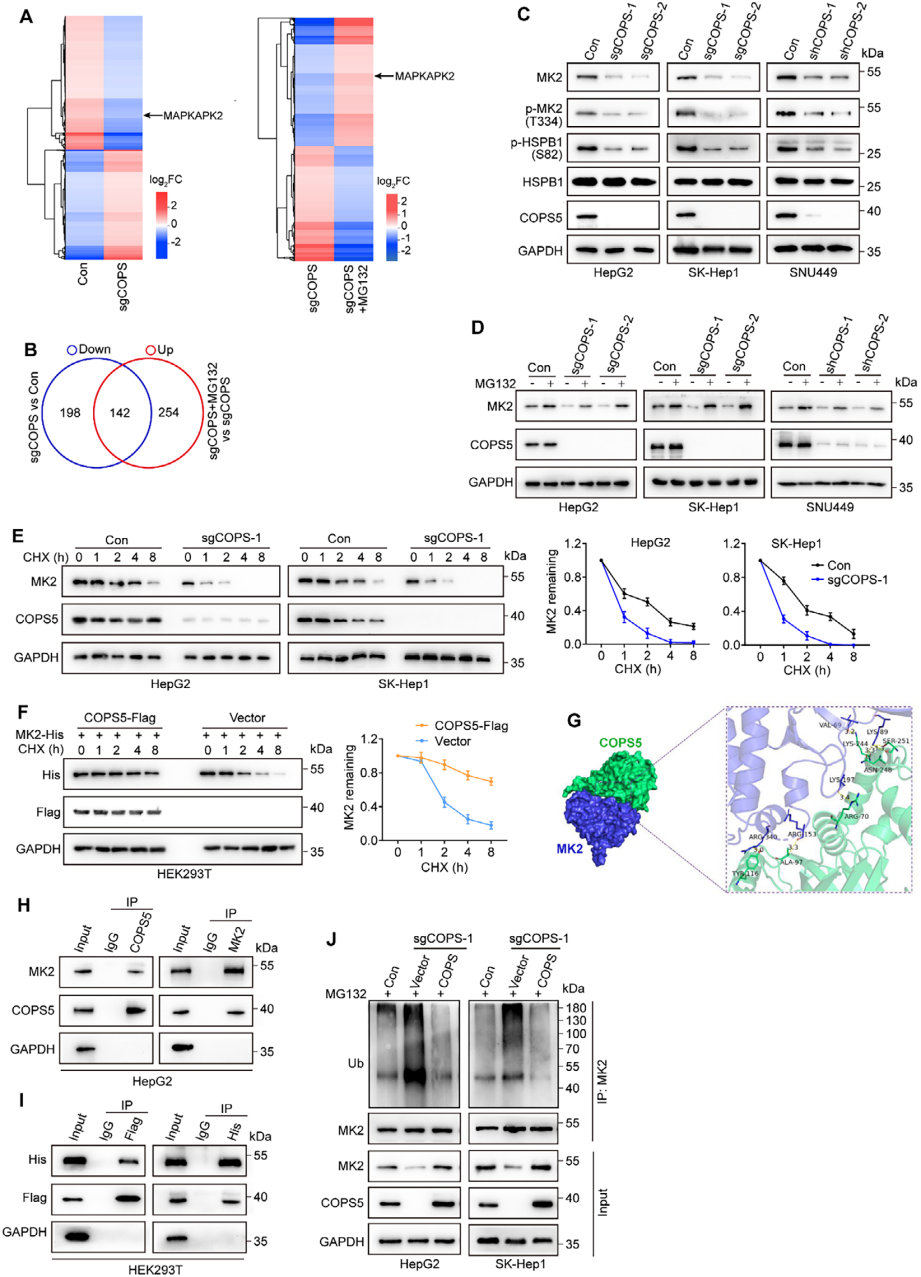
COPS5 stabilizes the MK2 protein and activates the MK2‐HSPB1 axis. A) Heat map depicting the differentially expressed proteins in COPS5‐KO versus control cells (left) and MG132‐treated versus untreated COPS5‐KO cells (right). B) Venn diagram illustrating proteins whose expression is downregulated upon COPS5 knockout but rescued by MG132 treatment. C) Western blots analysis of MK2, p‐MK2, p‐HSPB1, HSPB1, and COPS5 in COPS5‐KO/KD and control cells. D) Western blot analysis of MK2 and COPS5 in COPS5‐KO/KD and control cells treated with or without MG132 (20 µM). E,F) Cycloheximide (CHX) chase assay for MK2 protein stability in COPS5‐KO and control HCC cells (E) and HEK293T cells co‐transfected with the MK2‐His plasmid and COPS5‐Flag or control empty plasmid (F). G) Predicted interaction between COPS5 and MK2 by molecular docking. COPS5, green color; MK2, blue color. H) Reciprocal co‐IP between COPS5 and MK2 in HepG2 cells using anti‐COPS5 or anti‐MK2 antibody. I) Co‐IP of COPS5 and MK2 using anti‐Flag or anti‐His antibody in HEK293T cells co‐transfected with COPS5‐Flag and MK2‐His plasmids. J) Ubiquitination of MK2 was determined using IP/western blotting in COPS5‐KO, COPS5‐reexpressing, and control cells in the presence of MG132 (20 µM). Data are the mean ± SD. Statistical analysis was conducted using a two‐tailed *t*‐test.

To identify COPS5 substrates that mediate COPS5‐driven sorafenib resistance, we performed quantitative proteomic analysis in COPS5‐KO and control HepG2 cells. To rule out the possibility of non‐proteasomal degradation due to secondary effects, the proteasome inhibitor MG132 was administered to the cells, and additional proteomic analysis was conducted. As shown in **Figure** **4**A and Table S2, Supporting Information, 810 differentially expressed proteins (DEPs) were identified in COPS5‐KO cells compared with control cells, including 340 and 252 proteins with downregulated and upregulated expression, respectively. Gene Ontology (GO) enrichment analysis demonstrated that these DEPs were enriched in gene sets involved in the cell cycle, cell mitotic division, proteasomal protein catabolic processes, and ferroptosis‐related processes, such as oxidoreductase activity and aldo−keto reductase (NADP) activity (Figure S5A–C, Supporting Information). Among the 340 proteins downregulated by COPS5 knockout, the levels of 142 proteins, including MK2 (MAPKAPK2), were rescued by MG132 treatment (Figure 4B and Table S2, Supporting Information). MK2, a stress‐responsive kinase downstream of p38 MAP kinase, has been previously reported to promote the survival of cancer cells under various stimuli and chemotherapeutic resistance.^[^ ^21^, ^22^ ^]^ Importantly, its major substrate, HSPB1 (also called HSP27), negatively regulates ferroptosis by reducing intracellular iron accumulation and uptake.^[^ ^23^, ^24^, ^25^ ^]^ Therefore, we aimed to confirm whether MK2 was an essential downstream mediator of COPS5 function in ferroptosis resistance. We first assessed the changes in MK2 expression levels upon COPS5 modulation. As shown in Figure 4C and Figure S5D–F, Supporting Information, COPS5 depletion reduced, whereas COPS5 overexpression increased MK2 protein expression in HCC cells and xenograft tumors without affecting MK2 mRNA levels. Importantly, the downregulation of the MK2 protein in COPS5‐depleted cells was abolished by the proteasome inhibitor MG132 (Figure 4D), indicating that COPS5 regulates MK2 protein levels in a proteasome‐dependent manner. We evaluated the effect of COPS5 on MK2 stability and found that MK2 stability was lower in COPS5‐KO cells than in control cells (Figure 4E). To further confirm these results, the Flag‐tagged COPS5 plasmid was co‐transfected with the His‐tagged MK2 plasmid into HEK293T cells, and MK2 degradation was assessed. As expected, ectopic COPS5 expression blocked the degradation of exogenous MK2 (Figure 4F). To determine whether COPS5 stabilizes MK2 through deubiquitination, we first assessed whether COPS5 is associated with MK2. Molecular docking predicted the binding of COPS5 to MK2 (Figure 4G). Subsequent co‐immunoprecipitation (co‐IP) experiments also revealed that MK2 was detected in COPS5 immunocomplexes, while MK2 co‐immunoprecipitated with COPS5, suggesting a physiological interaction between COPS5 and MK2 (Figure 4H,I). The domain mapping assay showed that the C‐terminal domain of COPS5 (aa193–334) interacted with MK2, whereas the p38 MAPK‐binding domain (aa366–400) of MK2 interacted with COPS5 (Figure S6, Supporting Information). We then measured the effect of COPS5 on the ubiquitination of MK2 and observed that COPS5 KO in HCC cells led to a noticeable increase in MK2 ubiquitination; however, re‐expression of COPS5 offset this effect (Figure 4J). Taken together, these data support the hypothesis that COPS5 stabilizes MK2 via deubiquitination.

Unsurprisingly, COPS5 depletion reduced, whereas COPS5 overexpression induced the phosphorylation of MK2 (p‐MK2). Accordingly, the phosphorylation of its downstream effector HSPB1 (p‐HSPB1), which is required for ferroptosis suppression,^[^ ^23^, ^25^ ^]^ was decreased in COPS5‐depleted cells and restored in COPS5‐reexpressing cells, indicating MK2‐HSPB1 activation by COPS5 (Figure 4C; Figure S5D, Supporting Information).

We validated our findings by using clinical patient samples. The results of IHC staining showed that, compared with NATs, HCC samples presented increased levels of MK2 and p‐HSPB1 (Figure S7A, B, Supporting Information). Moreover, the high expression of MK2 or p‐HSPB1 predicted poor OS (Figure S7C, Supporting Information). More importantly, a noticeable correlation between COPS5 expression and MK2 or p‐HSPB1 expression was observed in HCC tissues (Figure S7D, Supporting Information), further supporting the notion that COPS5 induces the MK2‐HSPB1 signaling pathway. Additionally, MK2 expression positively correlated with p‐HSPB1 levels (Figure S7D, Supporting Information).

### 2.5 MK2‐HSPB1 Blunts Ferroptosis and Mediates COPS5‐Induced Sorafenib Resistance

**Figure 5:**
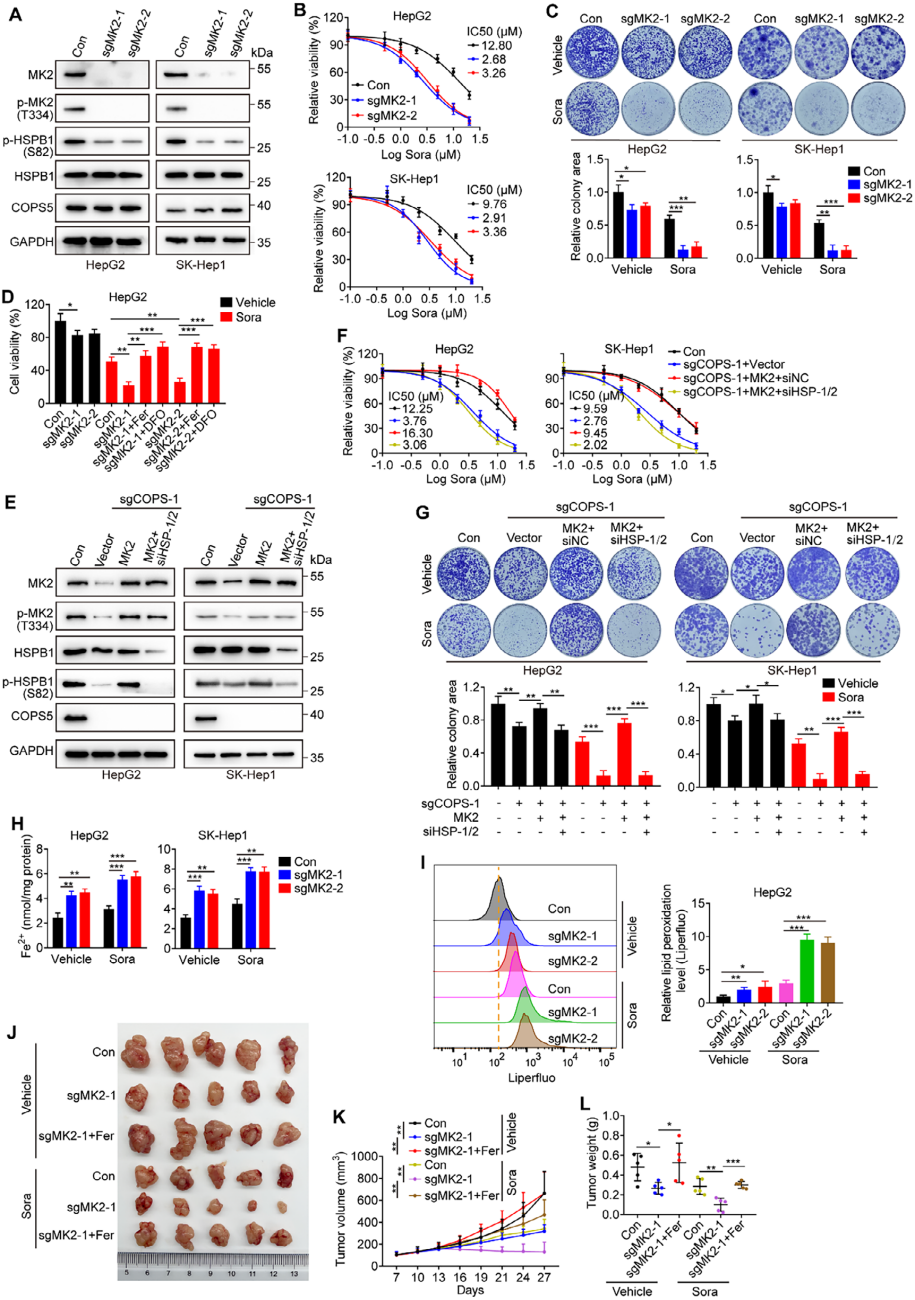
MK2/HSPB1 inhibits ferroptosis and mediates COPS5‐induced sorafenib resistance. A) Assessment of the protein levels of MK2, p‐MK2, p‐HSPB1, HSPB1, and COPS5 in the MK2‐KO and control cells. B) Viability of MK2‐KO and control cells treated with sorafenib for 72 h. C) Effects of MK2 knockout on the colony formation of HCC cells in the presence or absence of sorafenib. D) Viability of the MK2‐KO HepG2 cells cotreated with sorafenib and a ferroptosis inhibitor ferrostatin‐1 (1 µM) or deferoxamine (50 µM) for 48 h. E) Western blotting showing the protein levels of MK2, p‐MK2, HSPB1, p‐HSPB1, and COPS5 in COPS5‐KO cells transfected with the MK2 plasmid alone or in combination with HSPB1 siRNAs. F,G) COPS5‐KO cells were transfected with the MK2 plasmid alone or in combination with HSPB1 siRNAs, followed by exposure to sorafenib or the vehicle. Cell viability (F) and colony formation (G) assays were performed. H,I) Fe^2+^ content (H) and lipid peroxidation levels (I) of the MK2‐KO and control cells exposed to sorafenib or vehicle. J–L) BALB/c nude mice were subcutaneously injected with MK2‐KO or control HepG2 cells and then treated with vehicle or sorafenib alone or in combination with ferrostatin‐1 for 3 weeks (5 mice/group). Photographs of the tumors (J), tumor volumes (K), and tumor weights (L) are shown. Data are mean ± SD. Statistical analysis was conducted using a two‐tailed *t‐*test. *p < 0.05; ** *p* < 0.01; ***p < 0.001.

We explored the biological significance of MK2‐HSPB1 in regulating COPS5‐driven ferroptosis and sorafenib resistance. We generated MK2‐KO HepG2 and SK‐Hep1 cells and confirmed that p‐HSPB1 expression was attenuated in these cells (**Figure** **5**A). As expected, knockout of MK2 strikingly increased the cytotoxic effect of sorafenib/RSL3, decreasing the IC50 by three to sixfold, in parallel with the induction of cellular iron, lipid peroxidation, and MDA, as well as the typical morphological changes in mitochondria; these effects were reversed by ferroptosis inhibitors (Figure 5B–D, H and I; Figure S8A–D, Supporting Information). We also overexpressed MK2 in COPS5‐KO cells and found that MK2 overexpression rescued the effects of COPS5 KO on cell survival, as well as cellular iron, lipid peroxidation, and MDA levels after sorafenib treatment. Moreover, silencing of HSPB1 with small interfering RNA (siRNA) re‐sensitized MK2‐overexpressing cells to sorafenib‐induced ferroptosis (Figure 5E–G; Figure S8E–G, Supporting Information). Consistent with this, in vivo xenograft experiments showed that MK2 knockout reduced HCC growth and increased MDA and 4‐HNE levels in tumors. Furthermore, sorafenib‐induced tumor growth inhibition and ferroptosis were further enhanced after MK2 knockout, which was completely abolished by ferrostatin‐1 treatment (Figure 5J–L; Figure S8H, I, Supporting Information). Our data provide evidence that COPS5 triggers ferroptosis defense via the MK2‐HSPB1 pathway in HCC cells.

### 2.6 ATF4 Transcriptionally Activates COPS5 in Response to Sorafenib Treatment

**Figure 6:**
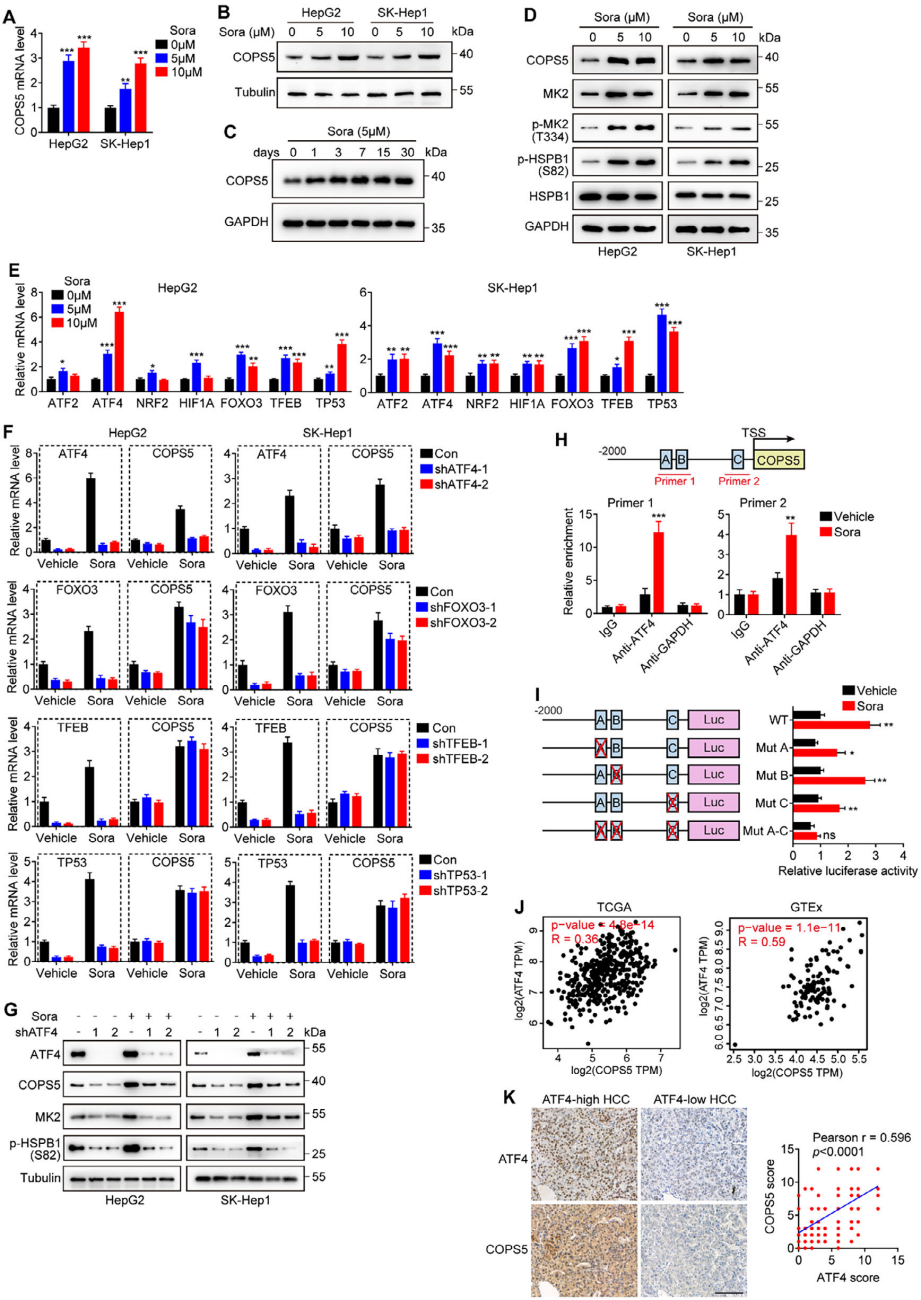
ATF4 transcriptionally upregulates COPS5 in response to sorafenib treatment. A, B) mRNA (A) and protein (B) expression levels of COPS5 in HepG2 and SK‐Hep1 cells following treatment with sorafenib for 24 h. C) Protein levels of COPS5 in HepG2 cells exposed to 5 µM sorafenib for 0, 1, 3, 7, 15, and 30 days. D) Western blots analysis of COPS5, MK2, p‐MK2, p‐HSPB1, and HSPB1 expression in HepG2 and SK‐Hep1 cells exposed to sorafenib. E) mRNA expression levels of ATF2, ATF4, NRF2, HIF1A, FOXO3, TFEB, and TP53 in HepG2 and SK‐Hep1 cells following treatment with sorafenib for 48 h. F) mRNA levels of COPS5, ATF4, FOXO3, TFEB, and TP53 in HepG2 and SK‐Hep1 cells transfected with the corresponding shRNAs in the presence or absence of 10 µM sorafenib. G) Effects of ATF4 knockdown on the protein levels of COPS5, MK2, and p‐HSPB1. H) ChIP‒qPCR assay of ATF4 enrichment on the COPS5 promoter in HepG2 cells treated with sorafenib or the vehicle. The primer pairs for the COPS5 promoter region used in the ChIP‒qPCR assay are shown. IgG and anti‐GAPDH antibodies were used as controls. I) Luciferase reporter assays of the COPS5 promoter region with either wild‐type (WT) or mutated ATF4 binding sites in HEK293T cells with or without sorafenib treatment. J) Correlations between COPS5 and ATF4 expression levels in the TCGA–LIHC and GTEx cohorts were analyzed using the GEPIA website. K) Representative IHC images showing ATF4 and COPS5 staining (left) and the correlation between COPS5 and ATF4 expression levels (right) in human HCC tissues. Scale bar: 150 µm. Data are the mean ± SD. Statistical analysis was conducted using a two‐tailed *t*‐test (A, E, F, H, and I) and a two‐tailed Pearson's test (J and K). ns, no significance; *p < 0.05; ** *p* < 0.01; ***p < 0.001.

We investigated whether COPS5 expression is regulated by sorafenib treatment. The findings revealed robust induction of COPS5 expression at both the mRNA and protein levels in HCC cells after 24 h of sorafenib treatment, with sustained upregulation of COPS5 observed following prolonged treatment for up to 30 days (**Figure** **6**A‒C). Sorafenib treatment strongly induced MK2 expression and phosphorylation of MK2 and HSPB1 (Figure 6D). Considering the role of sorafenib in regulating COPS5 mRNA levels, we evaluated whether COPS5 transcription was activated by sorafenib treatment. A luciferase reporter assay indicated that the luciferase activity of reporter constructs with 2 kb of the COPS5 promoter increased approximately threefold in HCC cells after exposure to sorafenib (Figure 6I), implying that the mechanism is likely transcriptional. Previous reports have shown that sorafenib can modulate several transcription factors, including ATF2,^[^ ^26^ ^]^ ATF4,^[^ ^27^ ^]^ NRF2,^[^ ^28^ ^]^ HIF1α,^[^ ^29^, ^30^ ^]^ FOXO3,^[^ ^31^ ^]^ TFEB,^[^ ^32^ ^]^ and p53,^[^ ^33^ ^]^ to affect cell survival or death. Next, we investigated whether these transcription factors could maintain the high expression of COPS5 in sorafenib‐treated HCC cells. We first sought to determine whether sorafenib treatment affected the expression of these transcription factors. The qRT‐PCR results confirmed that the expression levels of ATF4, FOXO3, TFEB, and p53 (TP53) consistently increased in HCC cells in response to different concentrations of sorafenib (Figure 6E). We then individually knocked down ATF4, FOXO3, TFEB, and p53 expression in sorafenib‐treated HCC cells to study their effects on COPS5 levels. ATF4 KD strongly suppressed sorafenib‐induced COPS5 upregulation, whereas KD of TFEB, p53, or FOXO3 had no or a marginal effect on COPS5 expression (Figure 6F). Consistently, ATF4 KD inhibited the activation of the MK2‐HSPB1 axis (Figure 6G). To determine whether ATF4 directly regulated COPS5 expression, we analyzed the chromatin immunoprecipitation (ChIP) sequencing results of ATF4 in the ENCODE database and found that ATF4 bound directly to the COPS5 promoter region in HepG2 cells (Figure S9A, Supporting Information). We then pinpointed the ATF4 binding sites in the COPS5 promoter using the websites JASPAR and HOCOMOCO, resulting in three predicted ATF4 binding sites at −1203 to −1215 (A), −1060 to −1072 (B), and −114 to −130 (C) in the COPS5 promoter (Figure S9B, Supporting Information). Chromatin immunoprecipitation with anti‐ATF4 antibodies, followed by qPCR, confirmed that ATF4 bound to the predicted binding sites in the COPS5 promoter region and that ATF4 enrichment in the COPS5 promoter was heavily elevated upon sorafenib treatment (Figure 6H). Furthermore, luciferase activity assessment revealed that when the predicted ATF4 binding site A or C of the COPS5 promoter was deleted, the effect of sorafenib on promoter luciferase activity was abolished, indicating that 2 of the 3 predicted ATF4 binding sites are essential for controlling COPS5 transcription (Figure 6I). Additionally, ATF4 KD reduced COPS5 promoter luciferase activity and offset the increase of luciferase activity induced by sorafenib (Figure S9C, Supporting Information). Taken together, our data indicate that sorafenib upregulates COPS5 expression via ATF4 induction.

Analysis of RNA sequencing (RNA‐seq) data from TCGA–LIHC and GTEx liver datasets revealed a positive correlation between ATF4 and COPS5 mRNA expression in both HCC samples and liver tissues (Figure 6J). Additionally, we observed that ATF4 mRNA levels were notably higher in HCC samples than in normal liver tissue samples and that patients with elevated ATF4 levels had shorter OS times than those with lower ATF4 levels (Figure S9D, E, Supporting Information). To validate the expression of ATF4 in human HCC, ATF4 protein levels were examined by IHC staining of human HCC specimens and NATs, as described above. As illustrated in Figure S9F,G, Supporting Information, the ATF4 protein was significantly overexpressed in HCC and was associated with shortened OS. Importantly, a strong positive correlation was observed between ATF4 and COPS5 protein expression in HCC tissues (Figure 6K), further supporting our finding that ATF4 positively regulates COPS5 expression.

### 2.7 MK2 Inhibitors in Combination with Sorafenib Synergistically Suppress HCC Progression

**Figure 7:**
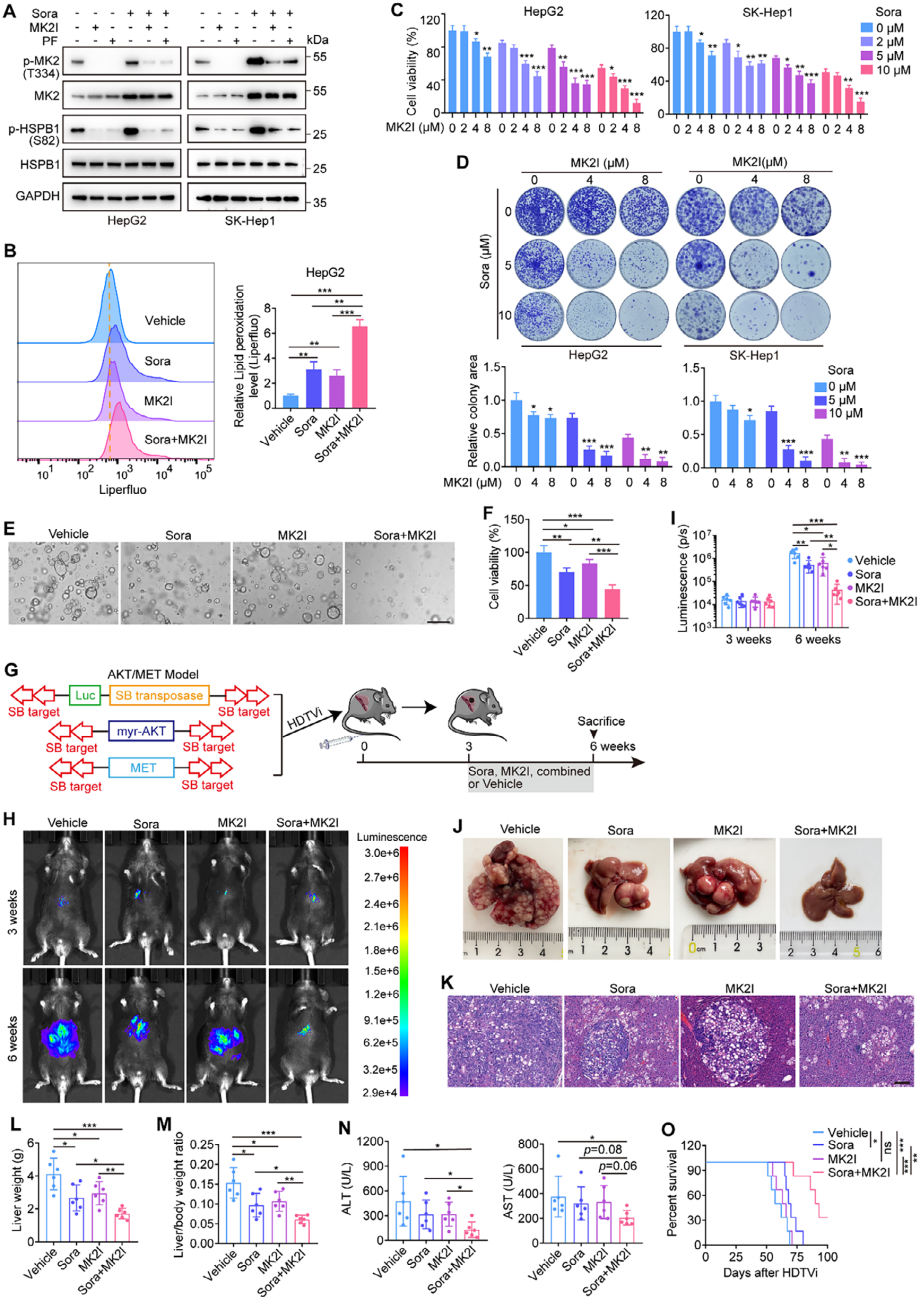
Combining MK2 inhibitors with sorafenib synergistically suppresses HCC growth. A) Protein levels of p‐MK2, p‐HSPB1, and HSPB1 in HepG2 and SK‐Hep1 cells treated with sorafenib (10 µM) alone or in combination with an MK2 inhibitor, 5 µM MK2 Inhibitor III (MK2I) or 5 µM PF3644022 (PF). B) Lipid peroxidation levels in HCC cells subjected to the indicated treatments. C) Viability of HepG2 and SK‐Hep1 cells cotreated with sorafenib (0, 2, 5, and 10 µM) and MK2 Inhibitor III (0, 2, 4, and 8 µM). D) Colony formation assay of HepG2 and SK‐Hep1 cells treated with a combination of sorafenib and MK2 Inhibitor III. E, F) Representative images (E) and viability (F) of PDOs treated with vehicle, sorafenib, MK2 Inhibitor III, or sorafenib plus MK2 Inhibitor III. Scale bar: 250 µm. G–O) The AKT/MET HCC models were established via hydrodynamic tail vein injection (HDTVi) of plasmids encoding the sleeping beauty transposase and transposons with the myr‐AKT gene and MET gene. 3 weeks after HDTVi of the plasmids, the mice were treated with vehicle, sorafenib, MK2 Inhibitor III, or sorafenib plus MK2 Inhibitor III for another 3 weeks (6 mice per group) (G). Representative bioluminescent images (H) and bioluminescence analysis results (I) of the mice in the four groups were shown. Representative photographs (J) and H&E staining (K) of livers, liver weights (L), liver body/weight ratios (M), and serum levels of ALT and AST (N) in mice from the four groups at 6 weeks after HDTVi of the plasmids were also shown. Scale bar: 150 µm (K). Kaplan‒Meier survival analysis of the survival of mice in the four groups were performed (O). Data are mean ± SD. Statistical analysis was conducted using a two‐tailed *t*‐test (B–D, F, I, L, M, and N) and a log‐rank test (O). ns, no significance; *p < 0.05; ** *p* < 0.01; ***p < 0.001.

Next, we studied the therapeutic efficacy of MK2 inhibitors combined with sorafenib for the treatment of HCC. As expected, treatment with two different MK2 inhibitors, MK2 Inhibitor III and PF3644022, abolished basal and sorafenib‐induced MK2/HSPB1 phosphorylation (**Figure** **7**A). Pharmacological inhibition of MK2 further enhanced cellular iron, lipid peroxidation, and MDA levels induced by sorafenib treatment, resulting in morphological alterations of mitochondria (Figure 7B; Figure S10A–C, Supporting Information). Importantly, combined treatment of HCC cells with MK2 inhibitors and sorafenib led to synergistic growth inhibitory activity (Figure 7C,D; Figure S10D, Supporting Information). To further determine the synergistic effects of the MK2 inhibitors and sorafenib, we established a patient‐derived organoid (PDO) model for in vitro studies. The combination of sorafenib and MK2 Inhibitor III synergistically attenuated the growth of HCC organoids (Figure 7E,F).

We subsequently studied combination therapy with sorafenib and MK2 Inhibitor III in an AKT/MET HCC mouse model generated via hydrodynamic tail vein injection (HDTVi) of the Sleeping Beauty Transposon system to overexpress myr‐AKT and MET (c‐MET). The mice were administered sorafenib and MK2 Inhibitor III daily, either alone or in combination, starting 3 weeks after HDTVi (Figure 7G). Over the course of a 3‐week treatment, we observed that, compared with monotherapy, the combination of sorafenib and MK2 Inhibitor III elicited a more pronounced inhibition of the tumor burden (Figure 7H–M). Serum alanine aminotransferase (ALT) and aspartate aminotransferase (AST) levels were substantially reduced only in the combination therapy group, suggesting a more effective reduction in disease severity (Figure 7N). Additionally, the combination therapy substantially prolonged the survival time of mice, whereas single‐drug treatments had a limited effect (Figure 7O). The body weights of mice across all groups were comparable, and the internal organs, including the heart, lungs, spleen, and kidneys, showed no morphological abnormalities (Figure S11, Supporting Information), suggesting good tolerability of the treatments in vivo. Together, these data suggest a synergistic antitumor effect when a combination of sorafenib and an MK2 inhibitor was used.

### 2.8 Inhibition of COPS5 with Curcumin Synergizes with Sorafenib to Attenuate HCC Development

**Figure 8:**
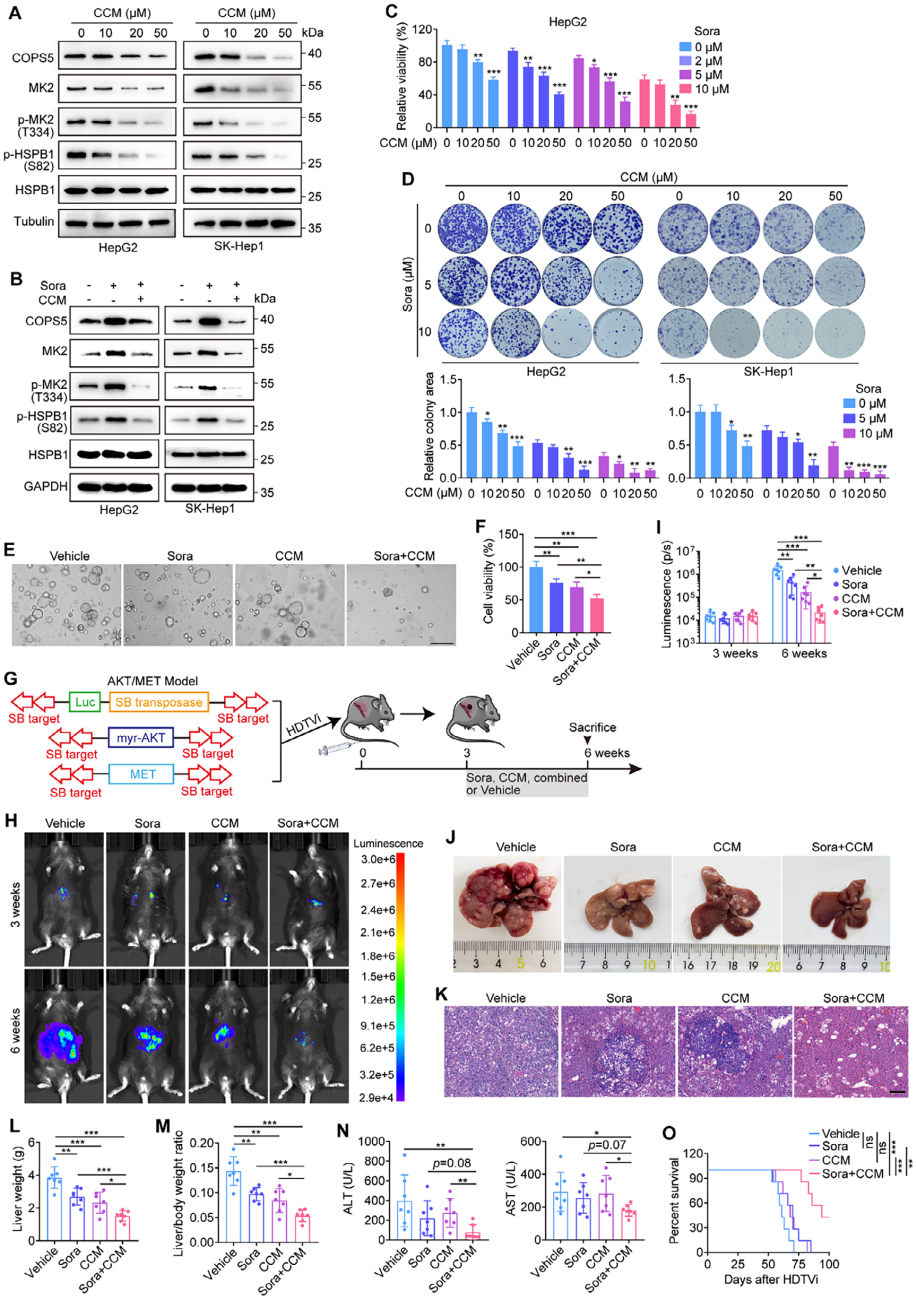
Curcumin synergizes with sorafenib to inhibit HCC progression. A) Western blots showing COPS5, MK2, p‐MK2, p‐HSPB1, and HSPB1 levels in HepG2 and SK‐Hep1 cells treated with increasing doses of curcumin (CCM). B) Protein levels of COPS5, MK2, p‐MK2, p‐HSPB1, and HSPB1 in HepG2 and SK‐Hep1 cells treated with 10 µM sorafenib alone or in combination with 20 µM curcumin. C) Viability of HepG2 cells cotreated with sorafenib (0, 2, 5, and 10 µM) and curcumin (0, 10, 20, and 50 µM). D) Colony formation assay for HepG2 and SK‐Hep1 cells treated with a combination of sorafenib and curcumin. E, F) Representative images (E) and viability (F) of PDOs treated with sorafenib, curcumin, the combination, or vehicle. Scale bar: 250 µm. G–O) The AKT/MET mice were treated with sorafenib, curcumin, the combination, or vehicle for 3 weeks (7 mice per group) (G). Bioluminescent images (H) and statistical analysis of bioluminescent tracking plots (I) of mice are shown. Representative photographs (J) and H&E staining (K) of livers, liver weights (L), liver body/weight ratios (M), and serum levels of ALT and AST (N) in mice from the four groups at 6 weeks after HDTVi of the plasmids are also shown. Scale bar: 150 µm (K). Kaplan‒Meier survival analysis of the survival of mice in the four groups was performed (O). Data are mean ± SD. Statistical analysis was conducted using a two‐tailed *t*‐test (C, D, F, I, L, M, and N) and a log‐rank test (O). ns, no significance; *p < 0.05; ** *p* < 0.01; ***p < 0.001.

To explore whether the pharmacological inhibition of COPS5 enhances the therapeutic efficacy of sorafenib against HCC, we used curcumin, a small‐molecule compound that has been demonstrated to inhibit not only COPS5‐associated deubiquitinase activity but also COPS5 expression,^[^ ^15^, ^34^ ^]^ for in vitro and in vivo studies. In this study, treatment of HCC cells with curcumin resulted in a dose‐dependent reduction in COPS5 and MK2 expression, as well as inactivation of the MK2‐HSPB1 axis (**Figure** **8**A). Moreover, curcumin combined with sorafenib treatment efficiently prevented the feedback accumulation of COPS5 elicited by sorafenib treatment, which was concomitant with the inhibition of MK2‐HSPB1 feedback activation (Figure 8B). Compared with sorafenib alone, curcumin co‐treatment caused stronger increases in the levels of cellular iron, lipid peroxidation, and MDA, as well as greater inhibition of cell survival. Additionally, curcumin alone modestly induced ferroptosis in cancer cells and suppressed cell growth (Figure 8C,D; Figure S12, Supporting Information), which was consistent with the results of previous studies.^[^ ^35^, ^36^ ^]^ Importantly, results from PDO model demonstrated that sorafenib and curcumin exhibited synergistic effects (Figure 8E,F).

We tested the antitumor efficacy of combining curcumin and sorafenib in vivo using an AKT/MET model. AKT/MET tumor‐bearing mice were administered sorafenib, curcumin, sorafenib plus curcumin, or vehicle (Figure 8G). When curcumin and sorafenib induced moderate or very mild antitumor effects, combined curcumin and sorafenib treatment led to increased efficacy compared with sorafenib or curcumin alone (Figure 8H–M), which indicated the synergistic effect of curcumin and sorafenib. Combination therapy also noticeably improved hepatic function and exhibited a survival advantage in tumor‐bearing mice, whereas monotherapy had mild or minimal effects on improving hepatic function and mouse survival (Figure 8N,O). Moreover, single or combined treatment with curcumin and sorafenib was well tolerated, as we did not observe any body weight loss or irregularities in internal organs, such as the heart, lungs, spleen, or kidneys (Figure S13, Supporting Information). Taken together, our results clearly demonstrated the critical role of curcumin in improving the efficacy of sorafenib treatment, suggesting a new combination strategy for the treatment of HCC.

## 3 Discussion

Sorafenib, a first‐line drug for advanced HCC, has limitations mainly due to drug resistance. However, effective therapeutic approaches to counter the resistance of cancer cells to sorafenib treatment are still lacking.^[^ ^37^ ^]^ Understanding the mechanisms underlying drug resistance is crucial for the design of improved therapeutic strategies. In the present study, using CRISPR/Cas9 knockout screening and functional validation, we identified COPS5 as a key mediator of survival following sorafenib treatment in HCC. Consistent with these findings, the amplification and overexpression of COPS5 were associated with a worse prognosis and poor response to sorafenib in patients with HCC. Mechanistically, COPS5 restrained ferroptotic cell death by activating the MK2‐HSPB1 pathway via its ability to stabilize MK2, whereas its expression was induced by sorafenib treatment via ATF4‐activated transcription. Furthermore, inhibition of COPS5 with curcumin or MK2 with MK2 inhibitors synergistically improved the anti‐HCC effects of sorafenib.

Ferroptosis has emerged as a significant process that affects drug sensitivity in cancer cells.^[^ ^38^ ^]^ The induction of ferroptosis represents a promising strategy to reverse tumor resistance to antitumor drugs.^[^ ^38^ ^]^ Numerous studies have characterized the function of sorafenib in ferroptosis induction.^[^ ^19^, ^20^ ^]^ However, a recent report has revealed that sorafenib does not necessarily trigger ferroptotic cell death in all tumor cell lines and that classic inhibitors of System Xc‐, such as sorafenib and erastin, can induce ferroptosis only in a subset of cancer cell lines.^[^ ^39^ ^]^ We propose that the intrinsic overexpression of COPS5 in many human cancers, including nasopharyngeal,^[^ ^40^ ^]^ liver,^[^ ^41^ ^]^ esophageal,^[^ ^42^ ^]^ stomach,^[^ ^43^ ^]^ pancreatic,^[^ ^44^ ^]^ lung,^[^ ^45^ ^]^ prostate,^[^ ^16^ ^]^ and breast^[^ ^46^ ^]^ cancers, may partially account for the resistance of cancer cells to ferroptosis inducers (e.g., sorafenib). Although the effect of COPS5 upregulation on the resistance to ferroptosis inducers in multiple types of cancer remains to be validated, our collective findings convincingly support the critical role of elevated COPS5 expression in rendering HCC insensitive to ferroptosis.

COPS5 is a multifunctional protein that regulates various cellular processes, including the cell cycle, cell proliferation, apoptosis, DNA repair, and signal transduction.^[^ ^47^ ^]^ Accumulating evidence suggests that COPS5 is an oncogene that contributes to cancer initiation and progression, as well as therapeutic resistance in various cancer cell types.^[^ ^14^ ^]^ Its best‐understood function is to regulate ubiquitin‐mediated protein degradation via deneddylation and deubiquitination, increasing the stability of target proteins.^[^ ^14^ ^]^ For instance, COPS5 prevents the ubiquitination and degradation of MDM2 and survivin, and promotes tumorigenesis.^[^ ^48^, ^49^ ^]^ Deubiquitination of Snail by COPS5 facilitates cancer cell invasion and metastasis.^[^ ^50^ ^]^ According to Bae et al.,^[^ ^51^ ^]^ COPS5 positively regulates VEGF transcription and angiogenesis by interacting with HIF‐1a and stabilizing the HIF‐1a protein under hypoxia. Recently, Lim et al.^[^ ^15^ ^]^ have reported that COPS5 deubiquitinates and stabilizes PD‐L1 and that inhibiting COPS5 with curcumin sensitizes cancer cells to immunotherapy. In accordance with the aforementioned findings, we discovered that COPS5 blocked the ubiquitination of MK2 and increased its stability in HCC cells, allowing HCC cells to escape ferroptosis, consequently enhancing sorafenib resistance. In contrast, COPS5 accelerates the degradation of several tumor suppressor proteins, such as p27,^[^ ^52^ ^]^ p57,^[^ ^41^ ^]^ and NCoR.^[^ ^46^ ^]^ For instance, COPS5 confers tamoxifen resistance by proteasomal degradation of NcoR in ERα‐positive breast cancer.^[^ ^46^ ^]^ These observations indicated that the regulatory role of COPS5 in protein stability is context‐dependent.

MK2 is a stress‐activated protein kinase that is phosphorylated by p38 MAPK under stressful conditions (e.g., hypoxia, oxidative stress, and radiation). Upon activation, it can phosphorylate HSPB1, a small heat shock protein, effectively protecting cells against stress injury.^[^ ^53^, ^54^ ^]^ Aberrant activation of this pathway is linked to human diseases such as cancer and inflammatory disorders.^[^ ^22^, ^55^, ^56^ ^]^ MK2/HSPB1 is upregulated and phosphorylated in some cancers and is associated with metastasis, progression, and response to therapy.^[^ ^53^, ^57^ ^]^ For example, enhanced MK2 levels in multiple myeloma drive tumor progression and drug resistance.^[^ ^58^, ^59^ ^]^ MK2/HSPB1 contributes to intestinal carcinogenesis and cancer progression, and inhibition of MK2 using small‐molecule inhibitors delayed intestinal carcinogenesis in an *Apc* ^min/+^ mouse model.^[^ ^60^ ^]^ Additionally, the activation of the MK2‐HSPB1 signaling pathway is a major survival mechanism that confers resistance to chemotherapeutic agents in pancreatic ductal adenocarcinoma.^[^ ^21^ ^]^ More importantly, HSPB1 reduces intracellular iron accumulation and uptake^[^ ^61^, ^62^ ^]^ and serves as a negative regulator of ferroptosis in tumors.^[^ ^23^, ^24^, ^25^ ^]^ Moreover, HSPB1 phosphorylation is required for its protective function in drug‐induced ferroptosis.^[^ ^23^, ^25^ ^]^ Herein, phosphorylation of HSPB1 was increased through COPS5‐mediated stabilization of MK2, which can mediate COPS5/MK2‐induced resistance to ferroptosis, suggesting the possibility of developing therapeutic strategies for improving the response to sorafenib by chemical inhibition of its upstream regulator COPS5 or MK2 as an alternative to its own inhibition, since there is a lack of inhibitors directly targeting HSPB1 phosphorylation. Indeed, we observed that targeting MK2 with small‐molecule inhibitors (MK2 Inhibitor III and PF3644022) or targeting COPS5 with curcumin strongly impaired the basal activation of the MK2‐HSPB1 signaling pathway, and the feedback activation of this pathway induced by sorafenib, augmented ferroptosis and synergized with sorafenib to attenuate the growth of HCC cells. Our study revealed that, as a single agent, MK2 Inhibitor III or curcumin attenuated HCC progression in AKT/MET mice, an autochthonous HCC model. MK2 Inhibitor III or curcumin combined with sorafenib synergistically inhibits HCC development without inducing additional toxicity. MK2 inhibitors have recently been shown to inhibit tumor proliferation and metastasis and increase the efficacy of other agents,^[^ ^21^, ^60^, ^63^ ^]^ and an oral MK2 inhibitor, ATI‐450, which prevents MK2 activation by inhibiting the p38‐MAPK‒MK2 interaction,^[^ ^64^ ^]^ is currently being evaluated in a phase 1/2 clinical trial for individuals with metastatic breast cancer (NCT06374459). Similarly, curcumin, a natural dietary supplement, works as an anticancer agent, as suggested by numerous preclinical studies across cancer types,^[^ ^65^ ^]^ and as a COPS5 inhibitor, as supported by multiple studies.^[^ ^15^, ^34^ ^]^ It has advanced into clinical trials for the treatment of colon cancer (NCT01490996), breast cancer (NCT01740323, NCT03072992), cervical cancer (NCT06080841), and pancreatic cancer (NCT00094445, NCT00192842), where it has been used either alone or in combination with chemotherapy and exhibits safe and tolerable antitumor activities. Thus, targeting the COPS5–MK2–HSPB1 axis in combination with sorafenib may be an effective and translatable therapeutic strategy for patients with HCC.

In conclusion, the current study demonstrates that COPS5, which is overexpressed in HCC in an amplification‐ and ATF4‐dependent manner, stabilizes MK2 through deubiquitination and, in turn, induces HSPB1 activation, protecting HCC cells from ferroptosis, thus promoting sorafenib resistance and tumor progression (Figure S14, Supporting Information). Our study provides a deeper understanding of the ferroptosis‐related mechanisms driving sorafenib resistance in HCC and suggests that the inhibition of the COPS5–MK2–HSPB1 axis is a potential therapeutic strategy for overcoming sorafenib resistance in HCC.

## 4 Experimental Section

### CRISPR/Cas9 Knockout Screening

The overall knockout screening workflow for this study is shown in Figure 1B. First, a stable Cas9‐expressing H22 cell line (H22‐Cas9) was established by infection of H22 cells with lentiCas9 viruses and selection with 8 µg mL^−1^ blasticidin for several days. The cells were subsequently transduced with the GeCKOv2.0 murine lentiviral library at a low multiplicity of infection (≈0.25) to ensure that most cells incorporated only a single sgRNA. After selection with 3 µg mL^−1^ puromycin for 5 days, the transduced cells were subcutaneously injected into BALB/c mice or BALB/c nude mice, which were then orally treated with vehicle or sorafenib (30 mg kg^−1^ daily) for 14 days. Tumors from all groups were collected, and genomic DNA was extracted using a DNA extraction kit. The sgRNA sequences were amplified using PCR, and the amplicons were subsequently subjected to high‐throughput sequencing by Novogene Technology (Beijing, China) to assess sgRNA abundance. Significantly depleted or enriched sgRNAs were identified from the comparison between sorafenib‐ and vehicle‐treated tumors using the MAGeCK algorithm.

### Statistical Analysis

Data are presented as the mean ± SD from either independent experiments or independent biological samples. All statistical analyses were performed using GraphPad Prism 8.0 (GraphPad Prism, USA). A two‐tailed *t*‐test was used to analyze the differences between groups. The Kaplan‐Meier method was used to generate survival curves, and the log‐rank test was used to compare differences between groups. Pairwise expression correlation analysis was conducted using Pearson's correlation test. Differences were considered statistically significant at *p* < 0.05.

### Ethics Approval Statement

This study involved human participants and was approved by the ethical standards of the Institutional Review Board at the Affiliated Cancer Hospital, Guangzhou Medical University. Informed consent was provided by all participants. Animal research was approved by the Institutional Animal Care and Use Committee of Guangzhou Medical University.

An additional experimental section is provided in the supplemental material.

## Conflict of Interest

The authors declare no conflict of interest.

## Author Contributions

Min Deng, Guo‐Hua Yang, and Chao Zeng are co‐corresponding authors. Ai‐Ling Luo, Wen‐Ying Zheng, and Qiong Zhang contributed equally to this study. Guarantor: Min Deng. Conception and design: Min Deng, Guo‐Hua Yang, and Chao Zeng. Development of methodology: Min Deng, Guo‐Hua Yang, Chao Zeng, Ai‐Ling Luo, and Wen‐Ying Zheng. Performed in vitro and in vivo experiments: Ai‐Ling Luo, Wen‐Ying Zheng, Qiong Zhang, Yan Yuan, Mei‐Qi Li, Kai Du, An‐Ran Gao, Li‐Jun Pei, Jie Xie, Wen‐Hao Chen, Xiu‐Zhu Guo, and Xiao‐Ran Yang. Clinical sample collection: Qiong Zhang and Long Zhang. Data analysis and interpretation: Ai‐Ling Luo, Wen‐Ying Zheng, and Qiong Zhang. Writing the manuscript and revisions: Min Deng, Guo‐Hua Yang, Chao Zeng, Ai‐Ling Luo, and Wen‐Ying Zheng. Funding acquisition and project administration: Min Deng, Long Zhang, and Yan Yuan. Study supervision: Min Deng, Guo‐Hua Yang, and Chao Zeng. Approved the manuscript: all authors.

## Supporting information



Supporting Information

Supplemental Table 1

Supplemental Table 2

Supplemental Table 3

Supplemental Table 4

Supplemental Table 5

## Data Availability

The information supporting the results of this research can be obtained from the corresponding author upon reasonable request. The CRISPR/Cas9 screening data are available from NCBI SRA (PRJNA1131614, https://dataview.ncbi.nlm.nih.gov/object/PRJNA1131614?reviewer=sk8agqu7sq8ul5e5tnpvj2ljal). The expression data for COPS5, ZRANB2, TSPAN17, HAUS1, KDELR3, and CDK5R1 from 67 patients with HCC who received sorafenib were obtained from GSE109211(https://www.ncbi.nlm.nih.gov/geo/query/acc.cgi?acc=GSE109211).^[^ ^13^ ^]^ RNA‐seq data from the TCGA–LIHC dataset were downloaded from TCGA database (https://portal.gdc.cancer.gov). Genomic alteration data of the TCGA–LIHC dataset were obtained from cBioPortal.^[^ ^66^ ^]^ Gene expression analysis of TCGA dataset was conducted using UALCAN.^[^ ^67^ ^]^ Correlations between gene expression levels and overall survival of patients and between ATF4 and COPS5 mRNA expression were assessed using GEPIA.^[^ ^68^ ^]^ Survival curves for sorafenib‐treated patients with HCC with high and low COPS5 expression were generated using the Kaplan–Meier plotter database.^[^ ^69^ ^]^ ChIP‐seq data were obtained from the ENCODE database.^[^ ^70^ ^]^ ATF4 binding sites in the COPS5 promoter region were predicted using JASPAR (https://jaspar.elixir.no/) and HOCOMOCO (https://molotool.autosome.org/).
