# Supplementary material for: COPS5 Triggers Ferroptosis Defense by Stabilizing MK2 in Hepatocellular Carcinoma
Source: Adv Sci (Weinh). 2025 Apr 8;12(22):2416360. doi: 10.1002/advs.202416360 (PMC12165036; doi:10.1002/advs.202416360)
Supplement: Supplementary file 1 — Supporting Information [file ADVS-12-2416360-s005.docx]

Supporting Information

**COPS5** **Triggers** **Ferroptosis Defense** **by Stabilizing MK2 in Hepatocellular Carcinoma**

Ai-Ling Luo, Wen-Ying Zheng, Qiong Zhang, Yan Yuan, Mei-Qi Li**,** Kai Du, An-Ran Gao, Li-Jun Pei, Jie Xie, Wen-Hao Chen, Long Zhang, Xiu-Zhu Guo, Xiao-Ran Yang, Chao Zeng *, Guo-Hua Yang *, Min Deng *

Table of Contents

[Experimental Section 2](#_Toc192657570)

[Supplementary Figures 9](#_Toc192657571)

[Supplementary references 22](#_Toc192657572)

# Experimental Section

**Antibodies and drugs**

The following antibodies and drugs were used in our research: anti-COPS5 antibody (sc-13157, Santa Cruz, USA), anti-MK2 antibody (sc-100393, Santa Cruz, USA), anti-p-MK2 antibody (sc-100393, Santa Cruz, USA), anti-HSPB1 (HSP27) antibody (2402, Cell Signaling Technology, USA), anti-p-HSPB1 (p-HSP27) antibody (2401, Cell Signaling Technology, USA), anti-MT1G antibody (CSB-PA17384A0Rb, Cusabio, China), anti-α-Tubulin antibody (2144, Cell Signaling Technology, USA), anti-ATF4 antibody (11815, Cell Signaling Technology, USA), anti-Ubiquitin antibody (58395, Cell Signaling Technology, USA), anti-GAPDH antibody (60004-1-AP, Proteintech, USA), anti-Flag antibody (66008-4-Ig, Proteintech, USA), anti-His antibody (ab18184, Abcam, USA), Goat anti-rabbit IgG (HRP) (C31460100, Invitrogen, USA), Goat anti-mouse IgG (HRP) (C31430100, Invitrogen, USA), anti-4-HNE (CAT. HY-P81208, MedChemExpress, USA), nonspecific rabbit IgG (PP64, Sigma‒Aldrich, USA), nonspecific rabbit IgG (PP54, Sigma‒Aldrich, USA), sorafenib (T0093L, TargetMol, USA), MK2 Inhibitor III (S6930, Selleckchem, USA), PF-3644022 (S8224, Selleckchem, USA), curcumin (T1516, TargetMol, USA), RSL3 (T3646, TargetMol, USA), erastin (T1765, TargetMol, USA), chloroquine (T8689, TargetMol, USA), deferoxamine (T124358, TargetMol, USA), ferrostatin-1 (S7243, Selleckchem, USA), necrostatin-1 (S8037, Selleckchem, USA), Z-VAD-FMK (S7023, Selleckchem, USA), puromycin (A1113803, Thermo Fisher Scientific, USA), blasticidin (A1113903, Thermo Fisher Scientific, USA), G418 (T6512, TargetMol, USA), MG132 (474790, Sigma‒Aldrich, USA), cycloheximide (239763, Sigma‒Aldrich, USA).

**Patient sample collection**

In total, 175 primary human HCC tissue samples and 76 normal adjacent tissue samples were collected at the Affiliated Cancer Hospital of Guangzhou Medical University (Guangzhou, China) between 2014 and 2019. Written informed consent was obtained from all participants, and the study was approved by the Institutional Review Board of the Affiliated Cancer Hospital, Guangzhou Medical University. Table S3 provides details of the patient samples.

**Cell culture**

HepG2, SK-Hep-1, Hep3B, PLC/PRF5, SNU449, Hepa1-6, MIHA, and HEK293T cell lines were purchased from the American Type Culture Collection. MHCC97-L and MHCC97-H were obtained from the Liver Cancer Institute of Fudan University. HUH-7, JHH-7, and QGS7701 were purchased from Jenniobio Biotechnology, whereas H22 cell line was obtained from Ubigene Biotechnology. The sorafenib-resistant cell subline HUH-7R was obtained from Bluefcell Biotechnology. All cell lines were cultured in DMEM (C11965500BT, Gibco, USA), RPMI-1640 (C12571500BT30, Gibco, USA), or MEM (C11875500BT, Gibco, USA) medium, supplemented with 10% fetal bovine serum (FBS) (04-001-1ACS, Biological Industries), in a humidified atmosphere of 5% CO_2_ at 37 °C. Cells were authenticated using short tandem repeat (STR) fingerprinting and tested for mycoplasma.

**Immunohistochemical staining**

Immunohistochemical staining was performed as previously described^[1]^. Briefly, tissue sections were deparaffinized, hydrated, subjected to microwave-assisted antigen retrieval, and blocked with 10% goat serum. The slides were then incubated with appropriate primary antibodies overnight at 4 °C and then with a secondary biotinylated antibody at room temperature for 20 min. Immunostaining was developed using the chromogen 3,3'-diaminobenzidine (DAB) (DAB-0031, Maixin, China). Staining intensity was graded as follows: 0, no staining; 1, weak staining; 2, moderate staining; and 3, strong staining. The percentage of positive cells was scored as follows: 0 (<5% positive cells), 1 (5–24% positive cells), 2 (25–50% positive cells), 3 (51–75% positive cells), and 4 (>75% positive cells). The final IHC score was calculated as follows: IHC score = intensity score × percentage score. Human samples with IHC scores ≥ 6 were considered to have high expression levels, whereas those with IHC scores < 6 were considered to have low expression levels.

**Vector** **construction, RNAi, and cell transfection**

To establish COPS5-KO or MK2-KO cell lines, sgRNAs targeting COPS5 or MK2 were annealed and ligated into the lentiCRISPR v2 vector (52961, Addgene). The plasmid containing sgRNA was co-transfected with the packaging vectors pMD2. G (12259, Addgene, USA) and psPAX2 (12260, Addgene, USA) into HEK293T cells using lipo^TM^ 8000 (C051, Beyotime, China) to produce the lentivirus. After 48 hours, lentiviral supernatants were collected and used to infect HCC cells. The cells were selected with 2–4 μg/mL puromycin for 7–10 days and then sorted into 96-well plates at a density of one cell per well via flow cytometry to allow them to proliferate for 2–3 weeks. Successful knockouts were evaluated using western blotting.

Full-length and truncated COPS5 sequences were subcloned into the pcDNA3.1(+)-C-Flag vector, whereas full-length and truncated MK2 sequences were subcloned into the pcDNA3.1(+)-C-6×His vector. These constructs were generated by Hanyi Biotech (China). To stably restore COPS5 expression in COPS5-KO cells, COPS5-KO cells were transfected with the COPS5-Flag plasmid using lipo^TM^ 8000 and selected in medium containing 400 mg/L G418. To generate cell lines stably overexpressing MK2, COPS5-KO cells were transfected with the MK2-His vector and incubated with 400 mg/L G418 for selection.

shRNAs targeting COPS5, MT1G, ATF4, FOXO3, TFEB, or TP53 were inserted into the pHBLV-U6-MCS-CMV-ZsGreen-PGK-PURO lentivirus vector (LV021, Hanbio, China) and packaged with the pMD2.G-psPAX2 system in HEK293T cells to generate lentiviral particles. After 48 hours, lentiviral supernatants were collected and used to infect the cells. siRNAs targeting human HSPB1 were obtained from Sangon (Shanghai, China) and transfected into the cells using Lipofectamine RNAiMAX (13778150, Invitrogen, USA). The sequences of sgRNAs, shRNAs, and siRNAs are listed in Table S4.

To generate luciferase reporter constructs for COPS5 promoter activity, the wild-type COPS5 promoter sequence (2 kb sequence upstream of the TSS) and its mutant counterparts (in which the predicted ATF4-binding sites were deleted) were chemically synthesized by Huada (Shenzhen, China) and inserted into the pGL3 basic vector (E1751, Promega, USA).

**Cell** **viability/growth,** **colony formation, and death assays**

For the viability and growth assay, cells (800–2000) were seeded in each well of 96-well plates and exposed to the specified treatments. After treatment, cell viability and growth were tested using the CCK-8 (CK04, Dojindo, Japan) assay. For the colony formation assay, cells were seeded at 1000–3000 cells per well in 12-well plates and treated with the indicated drugs for 9–14 days. Colonies were stained with 0.1% crystal violet (C0121, Beyotime, China), and the colony area in each well was quantified using ImageJ software. For cell death analysis, the cells were collected and stained with Annexin V-APC and 7-AAD (70-AT105, Multi Sciences, China) according to the manufacturer’s guidelines. Cell death was assessed using flow cytometry on a FACSCanto II Analyzer (BD Biosciences, USA).

**RNA** **isolation and RT-qPCR analysis**

Total RNA was isolated using the TRIzol reagent (15596018CN, Invitrogen, USA), and cDNAs were synthesized using a RevertAid reverse transcription kit (K1691, Thermo Fisher Scientific, USA). Gene expression was examined via the SYBR™ Green qPCR kit (4309155, Applied Biosystems, USA), and the results were normalized to the expression level of GAPDH. The primer sequences for each gene are listed in Table S5.

**Western blotting**

Cell samples were lysed in RIPA lysis buffer (P0045, Beyotime, China) supplemented with protease (04693132001, Roche, USA) and phosphatase (C0002, TargetMol, USA) inhibitors. After determining the protein concentration, protein lysates were separated by SDS-PAGE gel electrophoresis, and then transferred onto PVDF membranes. The membranes were probed with the designated primary antibodies overnight at 4 °C, followed by incubation with appropriate secondary antibodies at room temperature for 1 h. Band signals were identified using a Femto-sig ECL detection system (180-506, Tanon, China) and imaged using a 5200 chemiluminescent imaging system (Tanon, China).

**Co-immunoprecipitation**

Proteins were extracted from the indicated cells using IP lysis buffer (P0013, Beyotime, China) containing a protease inhibitor. The protein lysates were incubated with 1 μg of the appropriate antibodies overnight at 4 °C. Following incubation with Protein A/G magnetic beads (88802, Thermo Fisher Scientific, USA) for 1 h at room temperature, the protein-bead complexes were washed with IP lysis buffer and boiled in SDS loading buffer for 10 min. The resulting proteins were analyzed using western blotting.

**Chromatin immunoprecipitation**

The ChIP assay was conducted using a Chromatin IP Kit (9005, Cell Signaling Technology, USA) following the manufacturer's instructions. The cells were crosslinked for 10 min with 1% formaldehyde, and the reaction was quenched with glycine. Thereafter, the cells were washed with pre-chilled PBS and subjected to nuclear isolation. The nuclei were then incubated with micrococcal nuclease at 37 °C for 15 minutes, followed by the application of an appropriate amount of sonication, resulting in the digestion of chromatin DNA to lengths ranging from approximately 150 to 900 bp. Following centrifugation, the chromatin was immunoprecipitated with 2 μg of an anti-ATF4 antibody or the same amount of normal rabbit IgG at 4 °C overnight. The immunocomplexes were incubated with 30 µL of ChIP-grade Protein G magnetic beads at 4 °C for 2 hours. The beads were washed and eluted, after which the eluted chromatin was de-crosslinked, followed by protein digestion with proteinase K and RNA digestion with RNase. The resulting DNA was purified and analyzed using qPCR. The specific primers used for ChIP‒qPCR are listed in Table S5.

**Luciferase reporter assay**

The recombinant luciferase reporter plasmids containing the wild-type or mutant COPS5 promoter and the pRL-TK Renilla plasmid (used as an internal control, E2241, Promega, USA), with or without ATF4-shRNA, were co-transfected into HEK293T cells. After 24 h, the transfected cells were transfected and treated with either sorafenib (10 μM) or vehicle for another 24 hours and then examined using the Dual-Luciferase Reporter Assay System (E1910, Promega, USA), in accordance with the protocols provided by the manufacturer.

**Label-free quantitative proteomics** **analysis**

Proteins from COPS5-KO HepG2 cells with or without MG132 treatment and control HepG2 cells were extracted using RIPA lysis buffer supplemented with a protease inhibitor, reduced with dithiothreitol, and alkylated with chloroacetamide. Thereafter, the protein samples were digested with trypsin, and the peptides were collected and analyzed using an L-3000 high-performance mass spectrometer (RIGOL, China). Bioinformatics analysis of the raw mass spectrometric data was conducted using Proteome Discoverer 2.4 based on the UniProt database. Proteins with > 1.5-fold changes and *p* < 0.05 were considered to be differentially expressed. GO enrichment analyses were performed using the DAVID database (<http://david.ncifcrf.gov/>).

**Molecular docking**

The three-dimensional (3D) structures of the proteins COPS5 (PDB ID: 4D10) and MK2 (PDB ID: 1NXK) were downloaded from the RCSB Protein Data Bank. After 3D models of COPS5 and MK2 were generated using the MODELER protocol in Discovery Studio 4.5, a docking analysis of the COPS5 model with MK2 was performed using ZDOCK.

**Transmission electron microscopy**

The cells were fixed with 2.5% glutaraldehyde (P1126, Solarbio, China), washed, pre-embedded in agarose, and post-fixed with 1% buffered osmium. Following dehydration, the samples were embedded in resin, and ultrathin sections (60–80 nm thick) were prepared, stained with 2% uranyl acetate and subsequently with 2% lead citrate, and observed using an HT7800 transmission electron microscope (HITACHI, Japan).

**Patient-derived organoid model**

Fresh liver cancer tissues were washed with DMEM/F12 medium (11320033, Gibco, USA), cut into small pieces, and digested with collagenase IV (C4-28-100MG, Sigma-Aldrich, USA) at 37 °C for 30 min. After filtration with a 70-μm nylon cell strainer and centrifugation, the cell pellet was subjected to erythrocyte lysis (C3702, Beyotime, China). Cells resuspended in DMEM/F12 medium (11320033, Gibco, USA) were mixed with an equal volume of Matrigel (356231, Corning, USA), seeded into 24-well plates, and covered with MasterAim Liver Cancer Organoid Medium (10-100-296, AimingMed, China). Cells were cultured in a humidified incubator at 37 °C with an atmosphere of 5% CO_2_, and the medium was changed every 2–3 days.

**Animal experiments**

To investigate the impact of COPS5 and MK2 on sorafenib-induced ferroptosis in vivo, we subcutaneously injected COPS5-KO HepG2 cells or MK2-KO HepG2 and control HepG2 cells (5 × 10^6^ cells/mouse) into 7–8-week-old female BALB/c nude mice. When the tumors reached approximately 100 mm^3^ in volume, the mice were treated daily with the vehicle, sorafenib (30 mg/kg by oral gavage), or sorafenib (30 mg/kg by oral gavage) plus ferrostatin-1 (5 mg/kg, intraperitoneal administration) for 3 weeks. Tumor volumes were assessed at 2-day intervals and calculated using the following formula: volume = 0.5 × length × width^2^. Tumor weights were documented after the mice were sacrificed. To establish the AKT/MET HCC model, we hydrodynamically injected plasmids (20 μg) encoding sleeping beauty (SB) transposase and transposons with the myr-AKT gene and MET gene at a ratio of 1:10:10 in 2 mL of saline (0.9% NaCl) into the tail veins of 7–8-week-old male C57BL/6 mice. Three weeks after plasmid injection, the mice were orally treated daily with vehicle, sorafenib (30 mg/kg), sorafenib (30 mg/kg) plus MK2 Inhibitor III (20 mg/kg), or sorafenib (30 mg/kg) plus curcumin (30 mg/kg) for another 3 weeks. Tumor burden was monitored by luciferase signal intensity using an in vivo imaging system (AniView 600, BLT, China). The mice were sacrificed after 3 weeks of treatment, and the liver weight was measured as a surrogate for liver tumor burden. All animal studies were approved by the Institutional Animal Care and Use Committee of Guangzhou Medical University. The mice were housed in an animal room featuring a 12-hour light‒dark cycle, with a temperature of 23 ± 2 °C and humidity levels ranging from 30 to 70%.

**Measurement of serum AST and ALT**

After the mice were sacrificed, serum was immediately collected. Serum AST and ALT levels were measured using the standard clinical protocol of the Clinical Laboratory at the Affiliated Cancer Hospital, Guangzhou Medical University.

**Lipid peroxide** **and MDA assays**

Cellular lipid peroxide was detected by Liperfluo staining (L248, Dojindo, Japan). Cells were cultured in 12-well plates and treated with the indicated drugs. Liperfluo dye (2 μM) was added for 1 h at 37 °C, and the cells were digested with trypsin, collected, and analyzed using flow cytometry. The MDA content was measured using an MDA assay kit (S0131, Beyotime, China). The cells were lysed with lysis buffer and centrifuged. After incubation with thiobarbituric acid (TBA) at 100 °C for 15 min, the absorbance was then assessed at 532 nm via a microplate reader (Biotek, USA).

**Measurement of Fe^2+^**

Intracellular Fe^2+^ levels were measured using an Iron Assay Kit (I291, Dojindo, Japan) according to the manufacturer’s protocol. Briefly, the cells were harvested, incubated with Iron Assay Buffer at a volume ratio of 1:5 on ice for 5 min, ultrasonicated for 5 min, and centrifuged at 16,000 × *g* for 10 min. The supernatant samples were treated with Assay Buffer at a ratio of 20:1 (v/v) for 15 min at 37 °C and subsequently with an equal volume of Probe Solution for 1 h at 37 °C, after which the absorbance at 593 nm was detected.

# Supplementary Figures


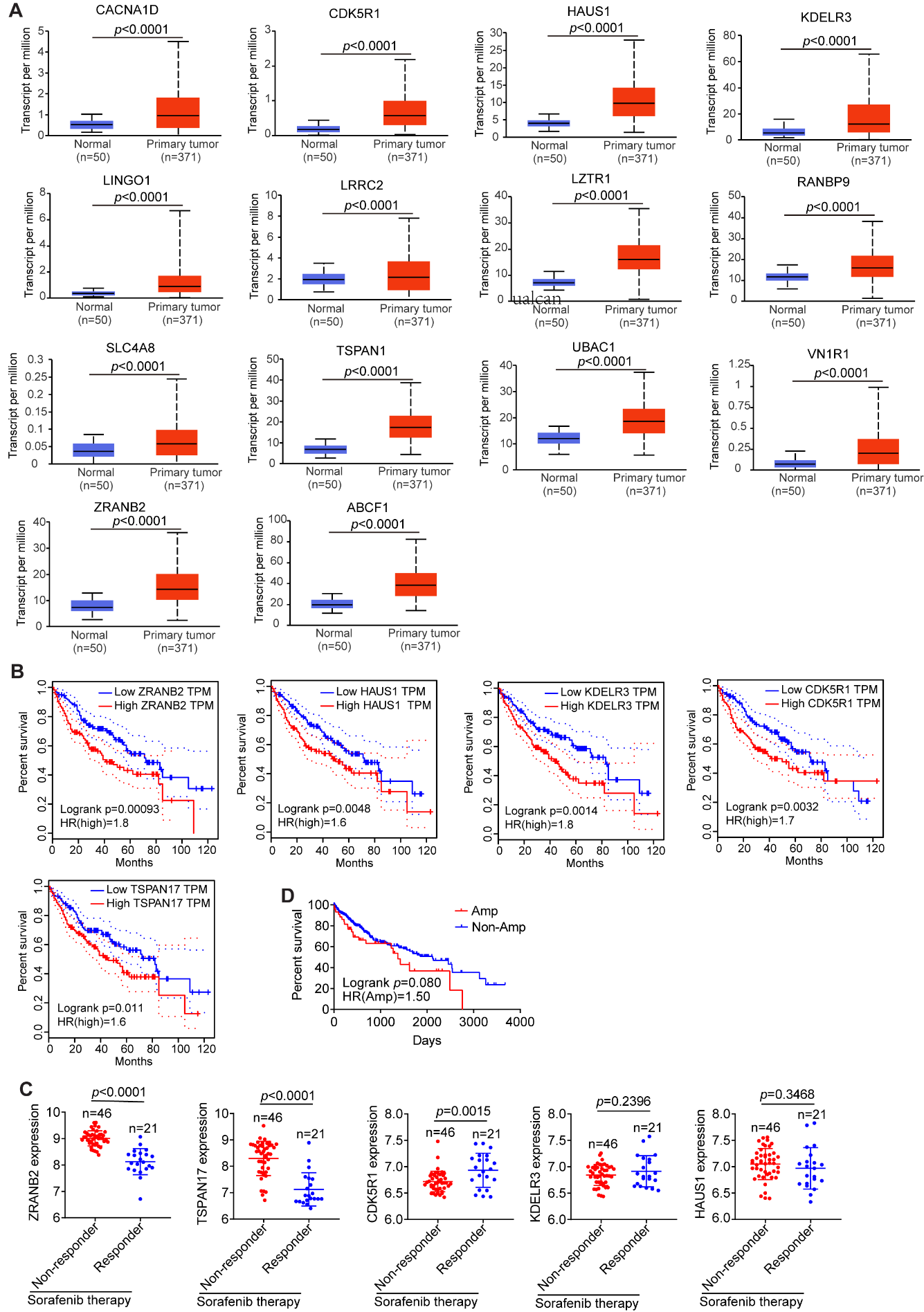


**Figure S1. Expression levels and clinical significance of the candidate genes identified by CRISPR/Cas9 screening.** A) The significant upregulation of genes in HCC samples relative to normal liver tissues from TCGA data is shown. B) The expression levels of ZRANB2, TSPAN17, HAUS1, KDELR3, and CDK5R1 are positively associated with poor overall survival in patients with HCC, according to TCGA data. C) The expression levels of the indicated genes in HCC tissues from sorafenib non-responders and sorafenib responders in the GEO dataset GSE109211^[2]^. D) Kaplan‒Meier survival curves of patients with HCC with or without COPS5 amplification were generated using TCGA–LIHC data via cBioPortal. Data are represented as mean ± SD. Statistical analysis was conducted using a two-tailed t-test (A and C) and a log-rank test (B and D).


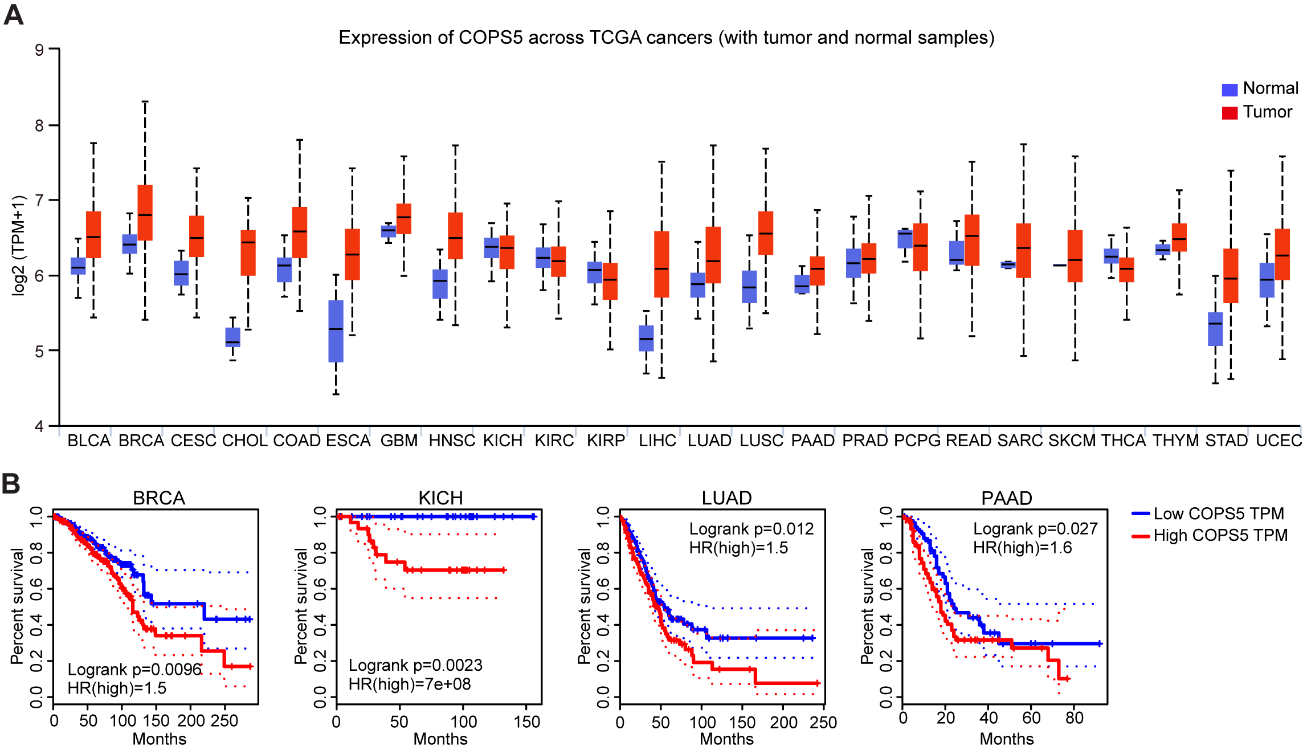


**Figure S2. TCGA pan-cancer analysis of COPS5 expression and its impact on survival.** A) Pan-cancer analysis of COPS5 expression in multiple cancer types. B) Overall survival analysis of high versus low COPS5 expression in patients from TCGA-BRCA, KICH, LUAD, and PAAD datasets.


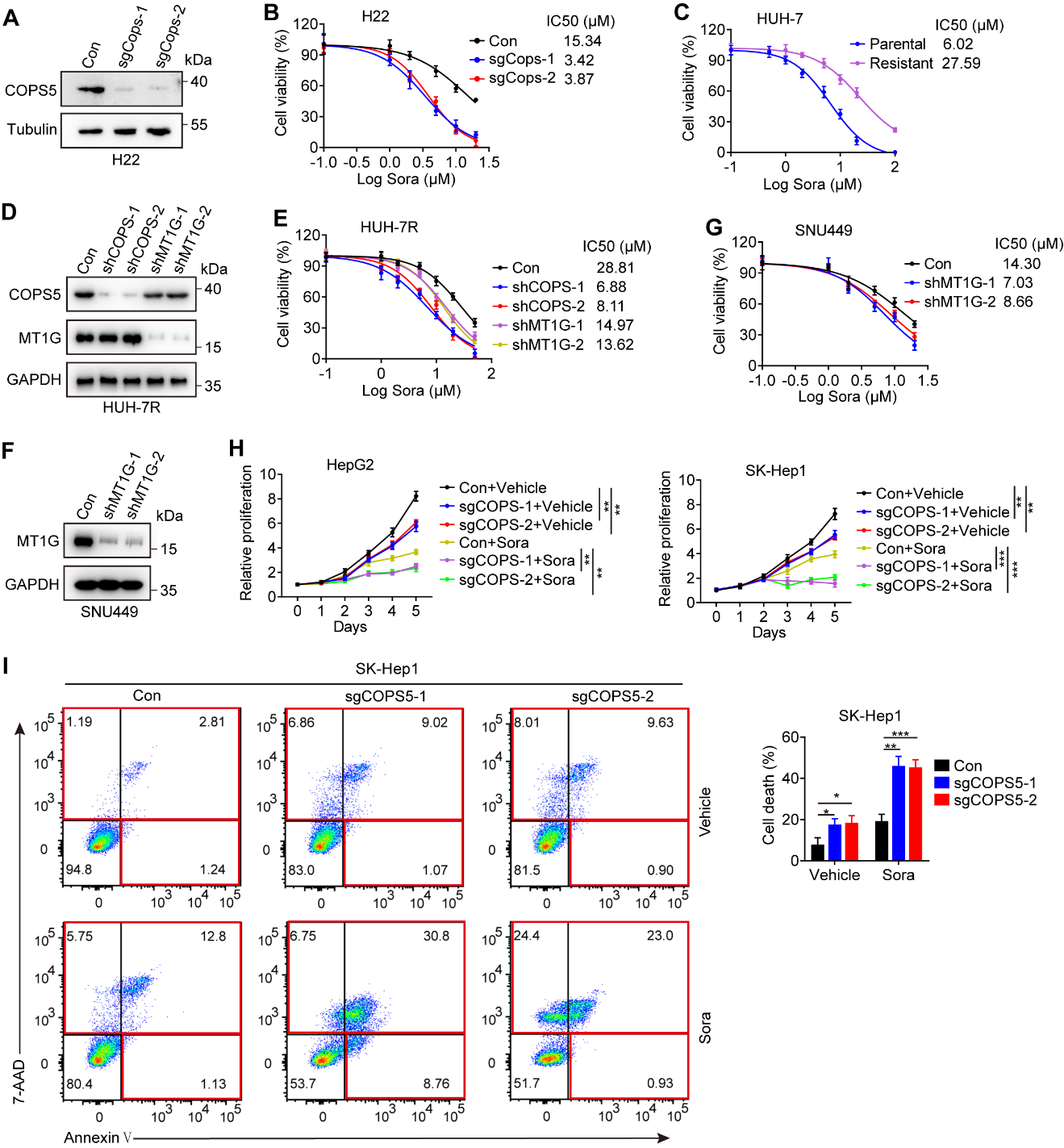


**Figure S3. COPS5 depletion synergizes with sorafenib to reduce** **cell proliferation and induce cell death in HCC.** A) COPS5 knockout in H22 cells was verified via western blotting. B) Viability of COPS5-KO and control H22 cells exposed to increasing concentrations of sorafenib for 72 h. C) Viability of HUH-7R and the parental HUH-7 cells exposed to increasing concentrations of sorafenib for 72 h. D) Western blots showing COPS5 and MT1G expression in COPS5-KD, MT1G-KD, and control HUH-7R cells. E) Cell viability of COPS5-KD, MT1G-KD, and control HUH-7R cells upon treatment with sorafenib for 72 h. F) MT1G knockdown in SNU449 cells was verified via western blotting. G) Viability of MT1G-KD and control SNU449 cells exposed to increasing concentrations of sorafenib for 72 h. H) The proliferation of COPS5-KO and control cells treated with vehicle or sorafenib (5 μM) was measured using a CCK-8 assay. I) Analysis of death in COPS5-KO SK-Hep1 cells treated with vehicle or sorafenib (10 μM). Data are represented as mean ± SD. Statistical analysis was conducted using a two-tailed t-test. **p* < 0.05, ** *p* < 0.01, ****p* < 0.001.


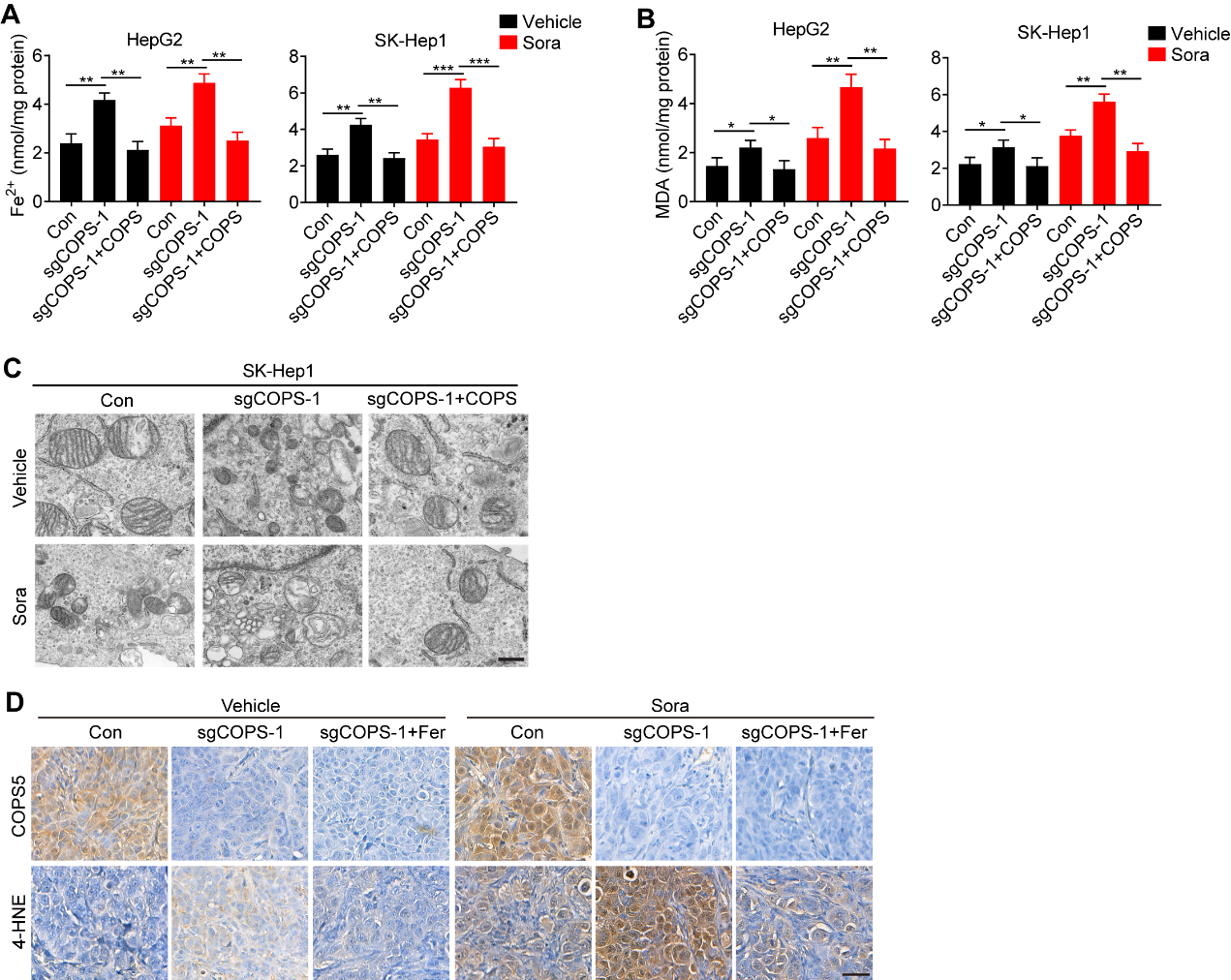


**Figure S****4. COPS5 inhibits ferroptosis in HCC cells.** A, B) Cellular Fe^2+^ (A) and MDA (B) levels were determined in the indicated cells exposed to vehicle or 10 μM sorafenib. C) Transmission electron microscopy images showing mitochondrial structures of COPS5-KO SK-Hep1 cells with or without COPS5 re-expression and control SK-Hep1 cells after treatment as indicated. Scale bar: 500 nm. D) IHC staining of COPS5 and 4-HNE in the xenograft tumors from BALB/c nude mice. Scale bar: 50 μm. Data are represented as mean ± SD. Statistical analysis was conducted using a two-tailed t-test. **p* < 0.05, ** *p* < 0.01, ****p* < 0.001.


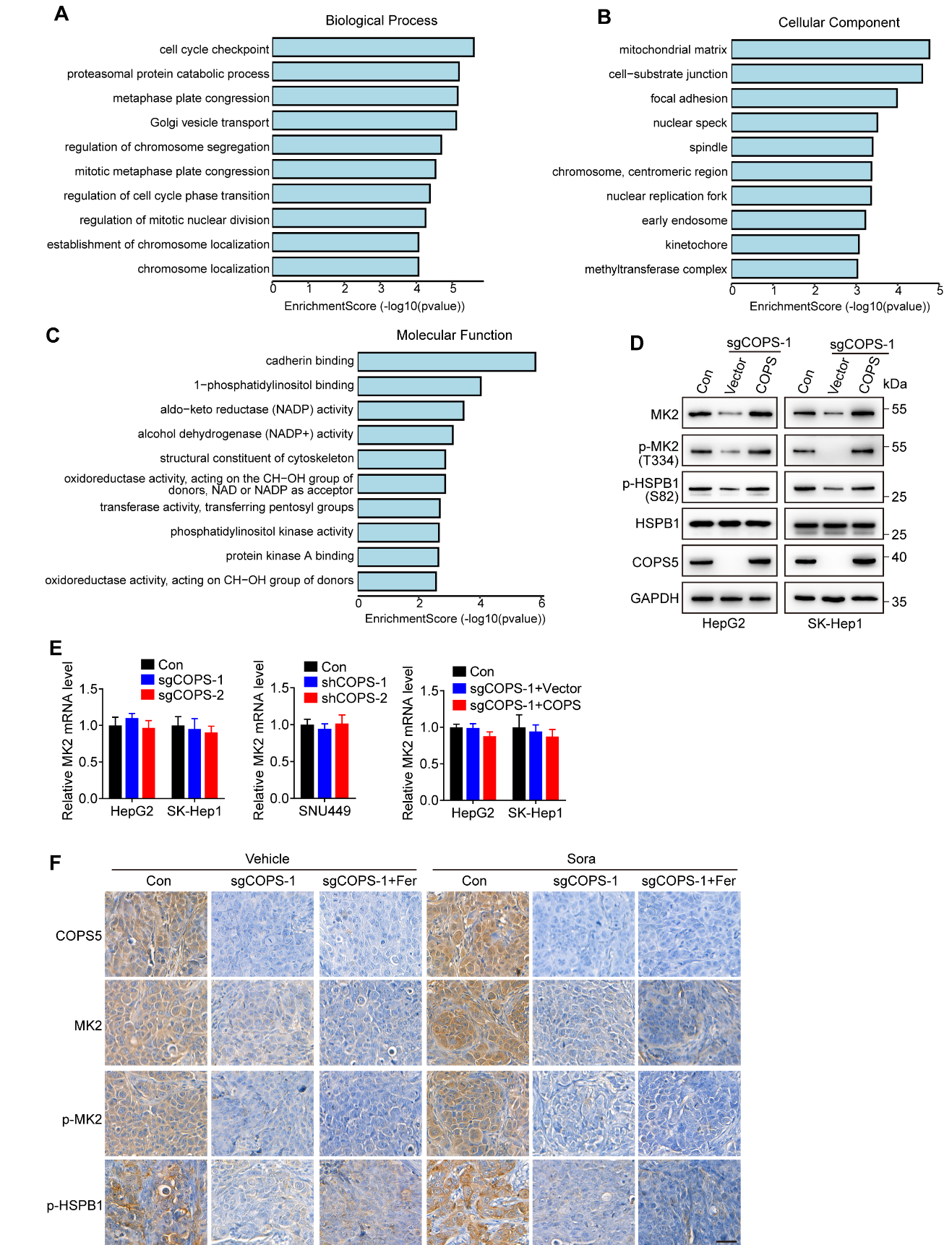


**Figure S5. COPS5 activates the COPS5–MK2–HSPB1 pathway.** A–C) Gene Ontology (GO) analysis of differentially expressed proteins in COPS5-KO vs. control cells, with the top 10 terms shown for biological process annotation (A), cellular component annotation (B), and molecular function annotation (C). D) Western blots showing MK2, p-MK2, p-HSPB1, HSPB1, and COPS5 levels in COPS5-KO cells with or without COPS5 re-expression and control cells. E) The mRNA levels of MK2 in COPS5-KO/KD and control cells. F) Representative IHC images of xenograft tumors showing COPS5, MK2, p-MK2, and p-HSPB1 staining. Scale bar: 50 μm. Data are represented as the mean ± SD. Statistical analysis was conducted using a two-tailed t-test.


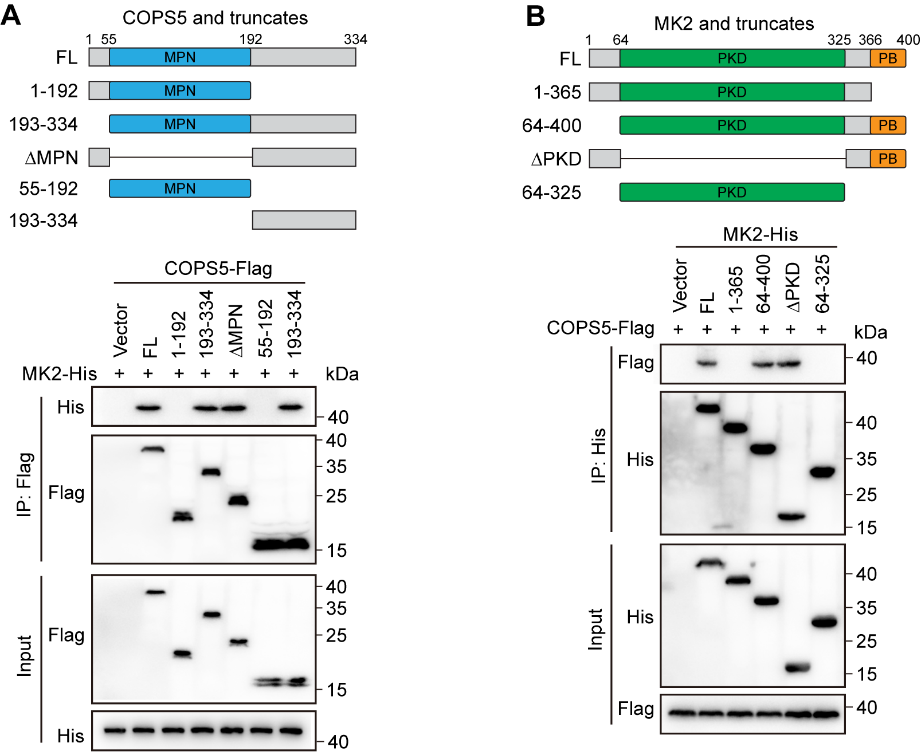


**Figure S6. Detection of the domains governing the interaction between COPS5 and MK2.** A) Schematic representation of various truncations of COPS5-Flag (upper) and co-IP assays to examine the interaction between MK2-His and different truncations of COPS5-Flag (lower). B) Strategy for constructing truncated MK2-His plasmids (upper) and co-IP analysis of COPS5-Flag interacting with different truncations of MK2-His (lower). FL, full-length; MPN, Mpr1p and Pad1p N terminus domain; PKD, Protein kinase domain; PB, p38 MAPK-binding domain.


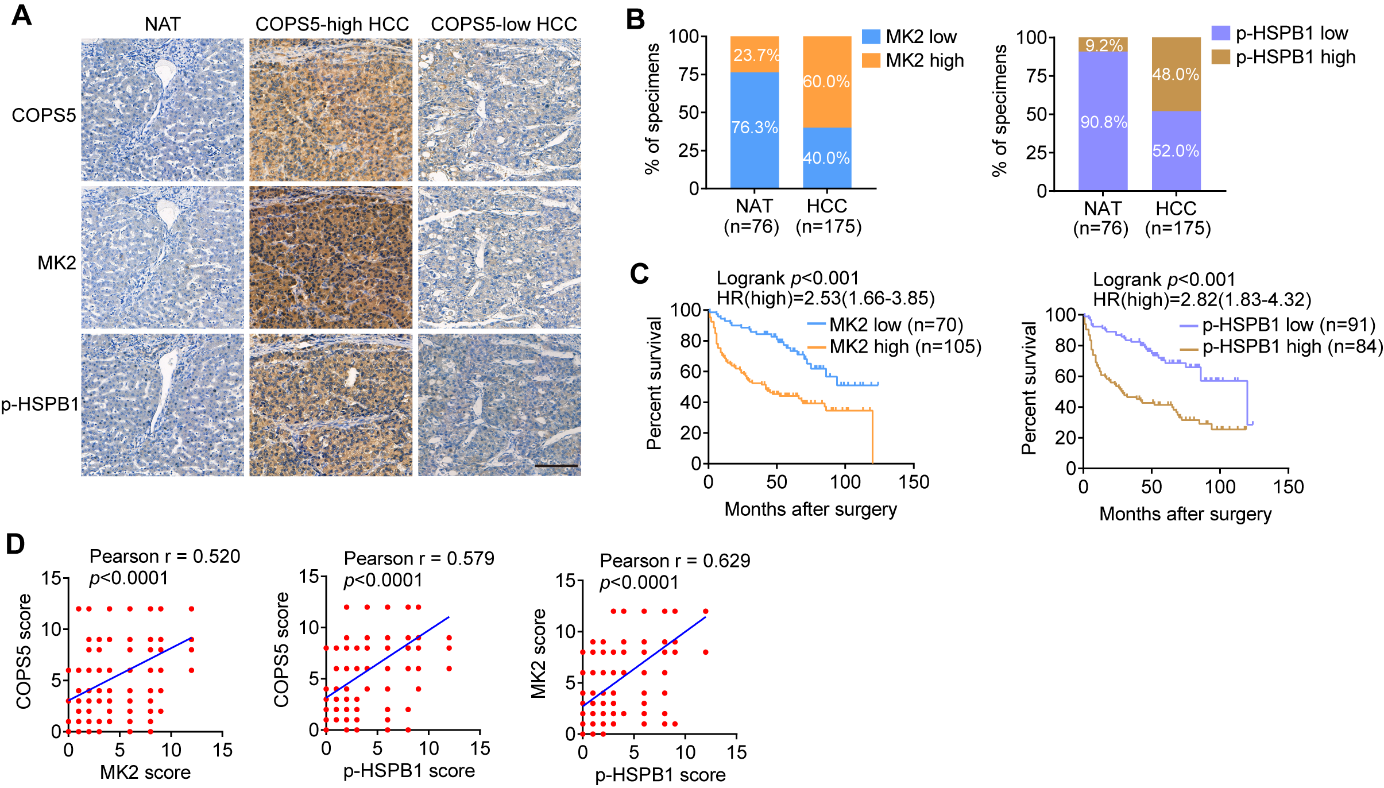


**Figure S7. COPS5 overexpression is associated with the** **activation of MK2–HSPB1 in clinical HCC samples.** A) Representative IHC images showing MK2 and p-HSPB1 levels in NATs and two HCC tissues with high and low expression of COPS5. Scale bar: 150 μm. B) Percentages of samples with high and low expression of MK2 or p-HSPB1. C) Kaplan‒Meier survival curves illustrating the survival of patients with HCC with high and low expression of MK2 or p-HSPB1. D) Pearson correlation analysis of COPS5 with MK2, COPS5 with p-HSPB1, and MK2 with p-HSPB1 in HCC tissues. Data are represented as the mean ± SD. Statistical analysis was conducted using a log-rank test (C) and a two-tailed Pearson’s test (D).


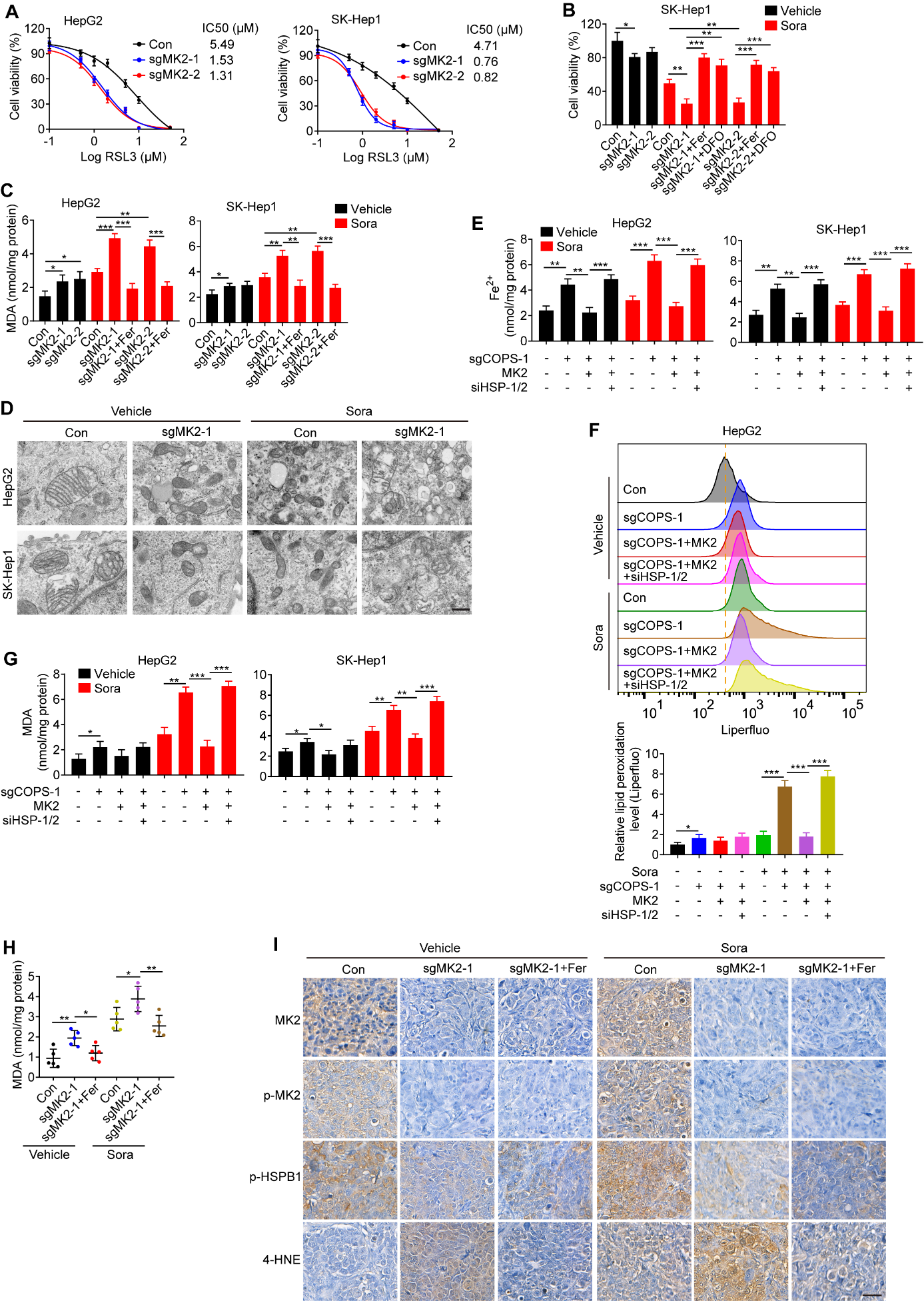


**Figure S****8. The COPS5–MK2–HSPB1 axis suppresses ferroptosis.** A) Cell viability of MK2-KO and control cells treated with increasing concentrations of RSL3 for 48 h. B) Viability of MK2-KO SK-Hep1 cells treated with vehicle, 1 μM ferrostatin-1, or 50 μM deferoxamine for 48 h. C) MDA levels in MK2-KO cells after treatment with sorafenib in the absence or presence of 1 μM ferrostatin-1. D) Transmission electron microscopy images showing morphological changes in the mitochondria of MK2-KO cells with or without sorafenib treatment. Scale bar: 500 nm. E–G) Fe^2+^ (E) and lipid peroxidation (F) and MDA (G) levels of COPS5-KO cells transfected with the MK2-His plasmid alone or in combination with HSP27 siRNAs in the absence or presence of sorafenib. H, I) MDA levels (H) and IHC staining of MK2, p-MK2, p-HSPB1, and 4-HNE (I) in xenograft tumors from BALB/c nude mice. Scale bar: 50 μm. Data are represented as the mean ± SD. Statistical analysis was conducted using a two-tailed t-test. **p* < 0.05, ***p* < 0.01, ****p* < 0.001.


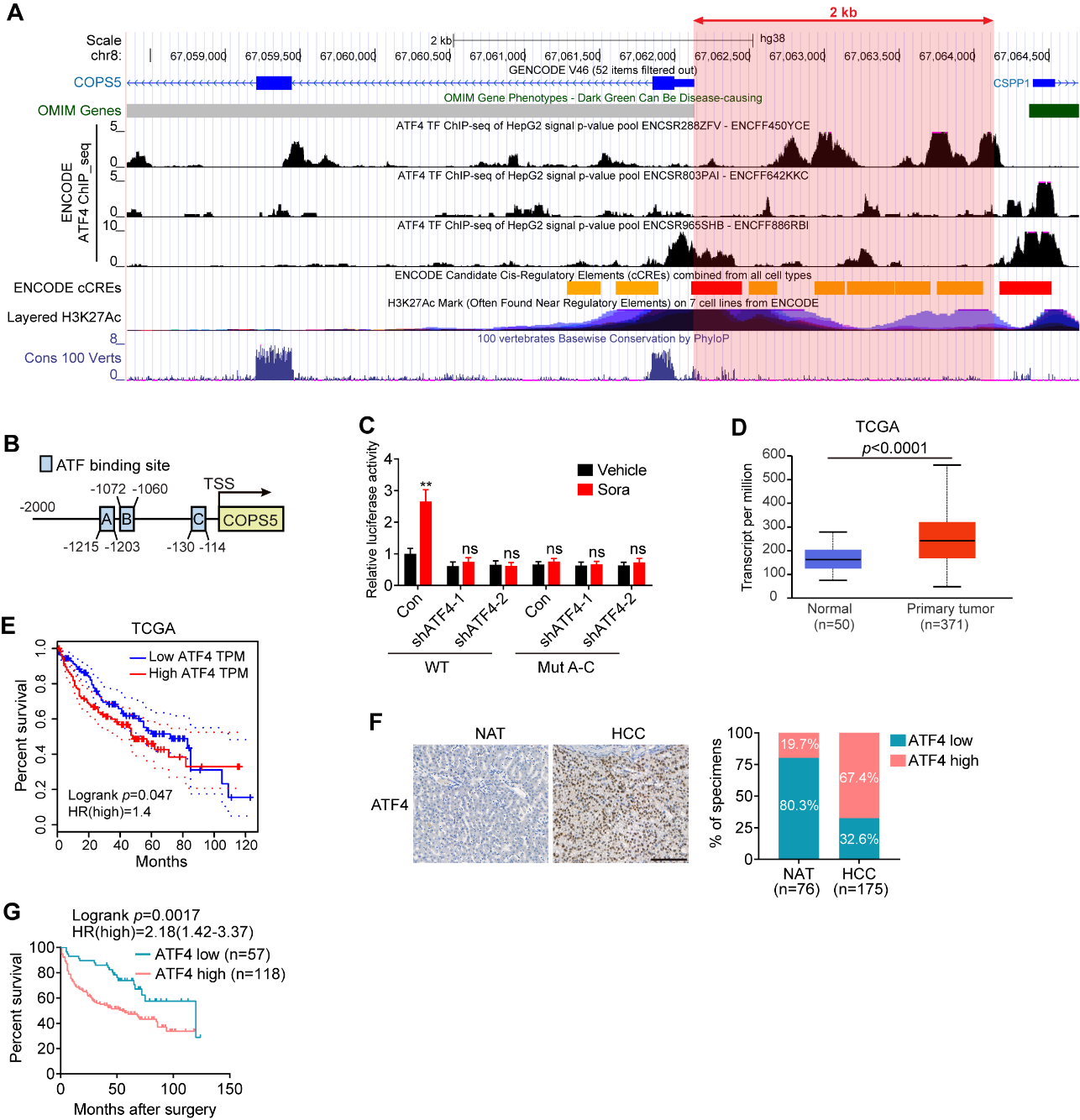


**Figure S****9. ATF4 binds to the COPS5 promoter region and is highly expressed in HCC tissues.** A) ENCODE ChIP-seq datasets showing ATF4 binding peaks on the COPS5 promoter region in HepG2 cells, accessible via the UCSC Genome Browser. The red box highlights the COPS5 promoter region. B) A schematic illustrating the three predicted ATF4 binding sites within the COPS5 promoter region. C) Luciferase reporter assays of COPS5 promoter region with either WT or mutated ATF4 binding sites in ATF4-KD and control HEK293T cells with or without sorafenib treatment. D) mRNA expression of ATF4 in the TCGA–LIHC cohort via the UALCAN platform. E) Association between ATF4 expression and HCC survival prognosis using TCGA–LIHC data via the GEPIA website. F) IHC analysis of ATF4 expression in HCC tissues and NATs. Scale bar, 150 μm. G) Kaplan‒Meier survival analysis of patients with high and low ATF4 levels. Data are the mean ± SD. Statistical analysis was conducted using a two-tailed t-test (C and D) and a log-rank test (E and G).


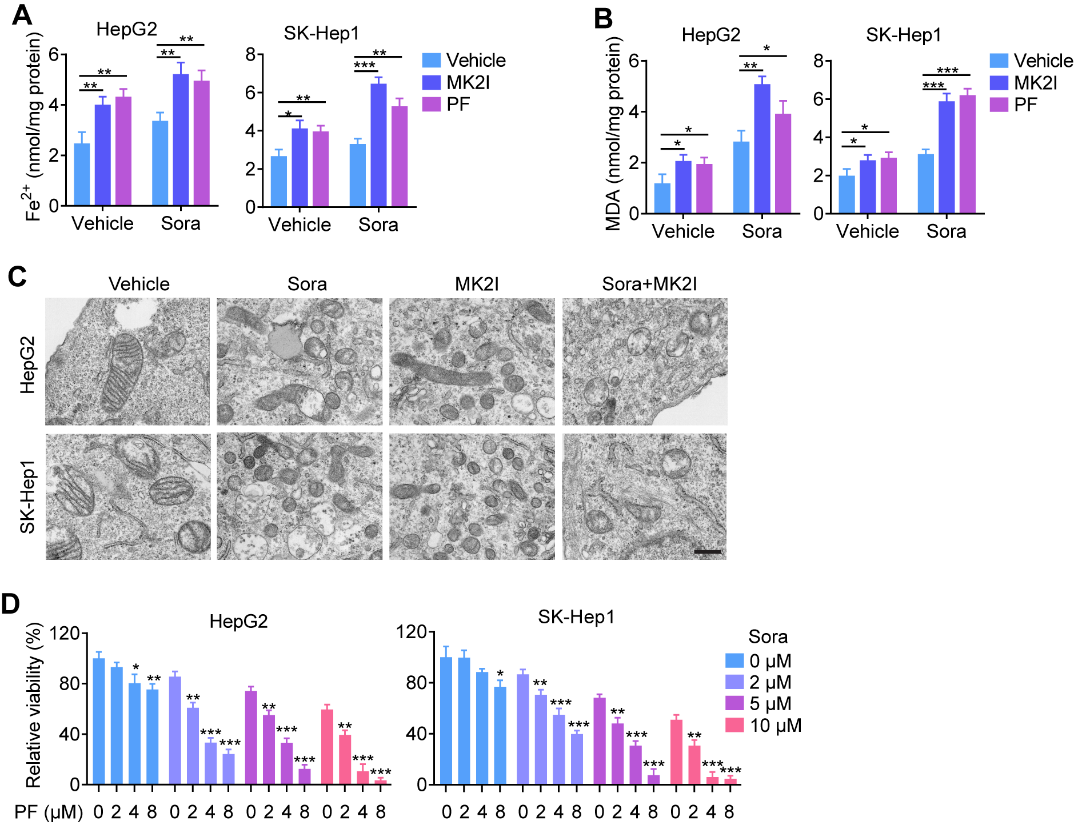


**Figure S10. MK2** **inhibitors combined with sorafenib synergistically** **induce ferroptosis in HCC cells.** A, B) Fe^2+^ (A) and MDA (B) levels of HCC cells treated with 10 μM sorafenib alone or in combination with MK2 Inhibitor III (5 μM) or PF-3604422 (5 μM). C) Transmission electron microscopy analysis of mitochondrial structures in HepG2 and SK-Hep1 cells treated with sorafenib, MK2 Inhibitor III, a combination or vehicle. Scale bar, 500 nm. D) Viability of HepG2 and SK-Hep1 cells cotreated with sorafenib and PF-3604422. Data are the mean ± SD. Statistical analysis was conducted using a two-tailed t-test. **p* < 0.05, ***p* < 0.01, ****p* < 0.001.


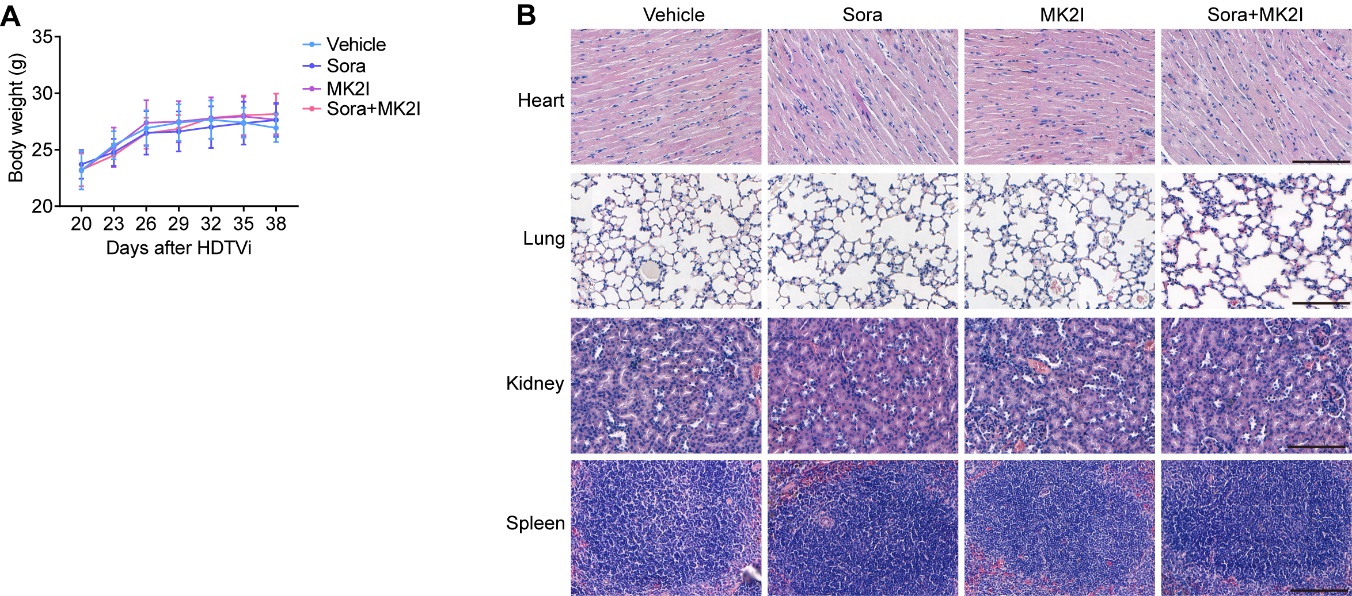


**Figure S11. Effect of combined treatment with the MK2** **Inhibitor III/sorafenib on the body weight and internal organs of AKT/MET mice.** A) Body weight curves of AKT/MET mice treated with sorafenib, MK2 Inhibitor III, a combination or vehicle. B) Representative H&E images of the heart, lung, kidney, and spleen tissues from mice treated with sorafenib, MK2 Inhibitor III, a combination or vehicle. Scale bar: 150 μm. Data are the mean ± SD.


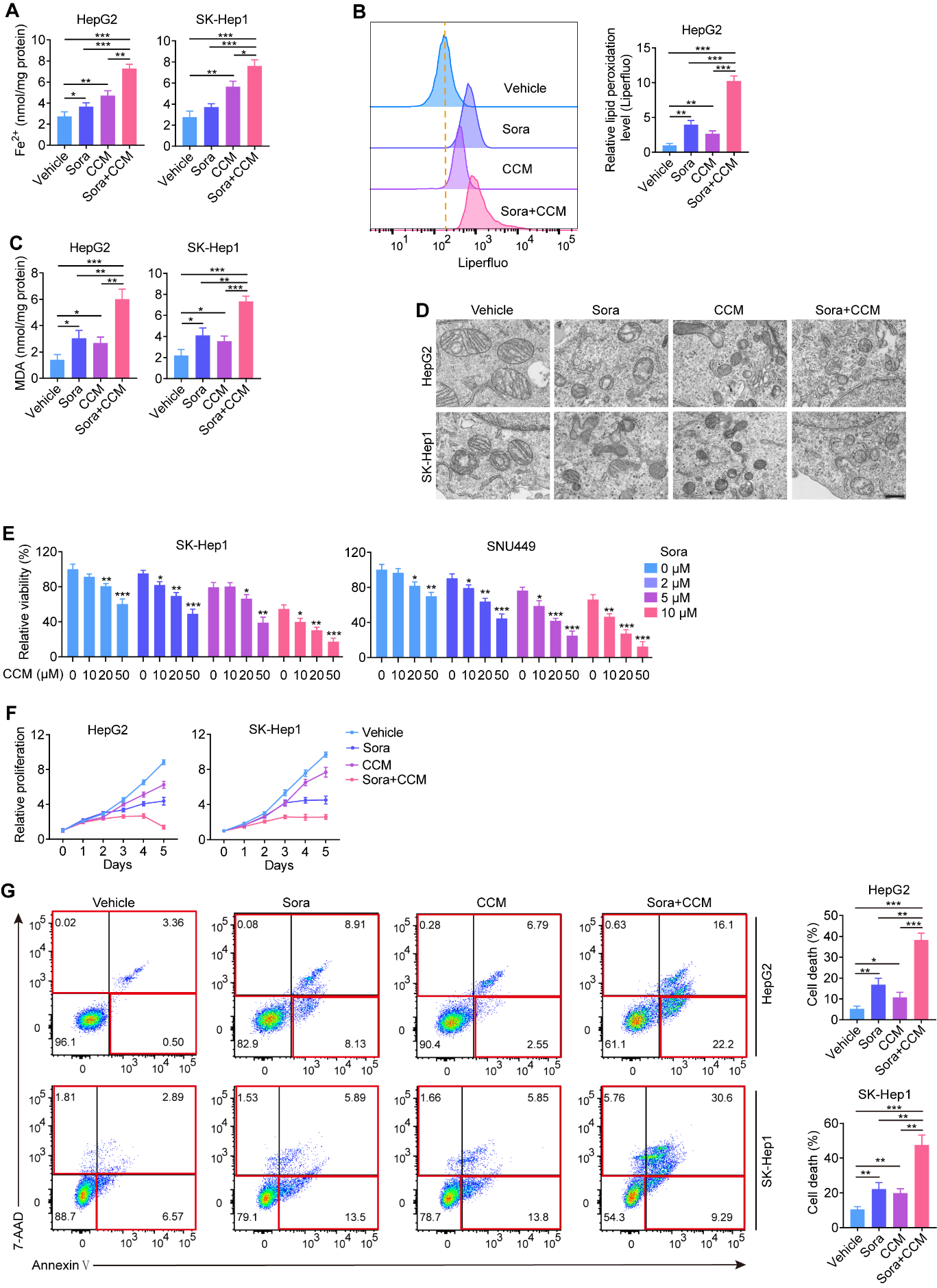


**Figure S12. Curcumin enhances cell death induced by sorafenib** **in HCC cells.** A–C) Fe²⁺(A), lipid peroxidation (B), and MDA (C) levels of HCC cells treated with sorafenib, curcumin, or a combination. D) Transmission electron microscopy analysis of mitochondrial structures in HepG2 and SK-Hep1 cells treated with sorafenib, curcumin, or a combination. E) Viability of SK-Hep1 and SNU449 cells cotreated with sorafenib and curcumin for 48 h. F, G) Proliferation (F) and cell death (G) levels of HCC cells treated with sorafenib, curcumin, or a combination. Data are the mean ± SD. Statistical analysis was conducted using a two-tailed t-test. **p* < 0.05, ***p* < 0.01, ****p* < 0.001.


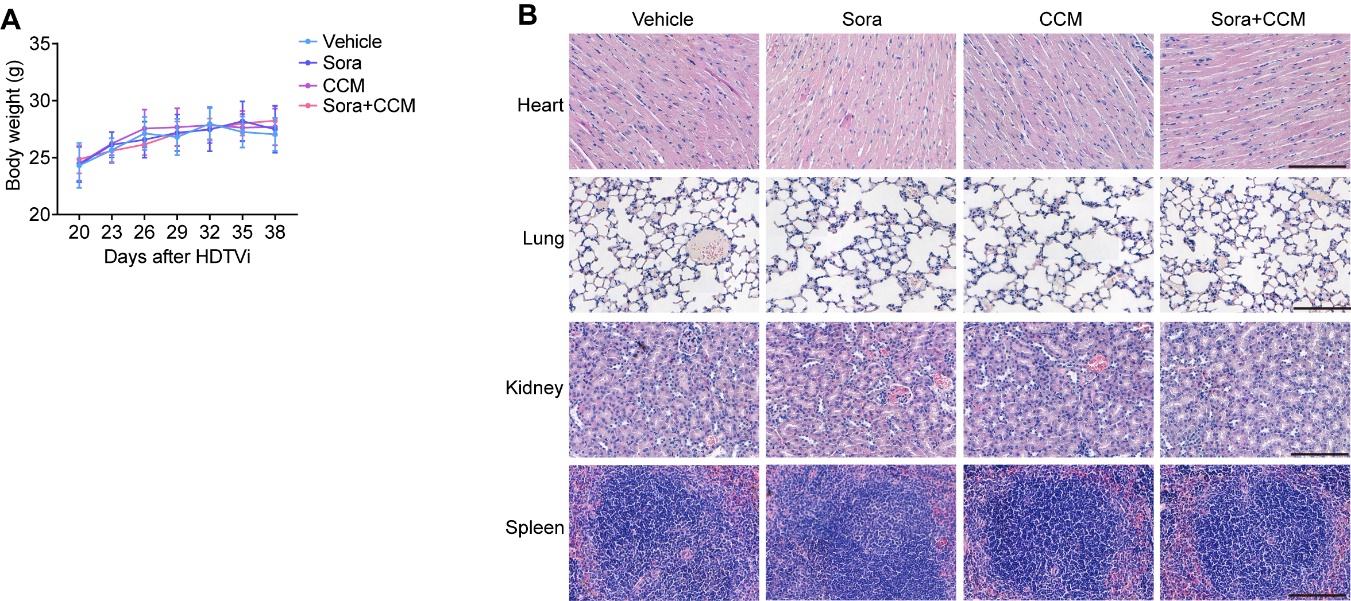


**Figure S13. Effects of curcumin combined with sorafenib on the** **body weight and internal organs of AKT/MET mice.** A) Body weight curves of AKT/MET mice treated with sorafenib, curcumin, a combination, or vehicle. B) Representative H&E images of the hearts, lungs, kidneys, and spleens from AKT/MET mice treated with sorafenib, curcumin, a combination, or vehicle. Scale bar: 150 μm. Data are represented as the mean ± SD.


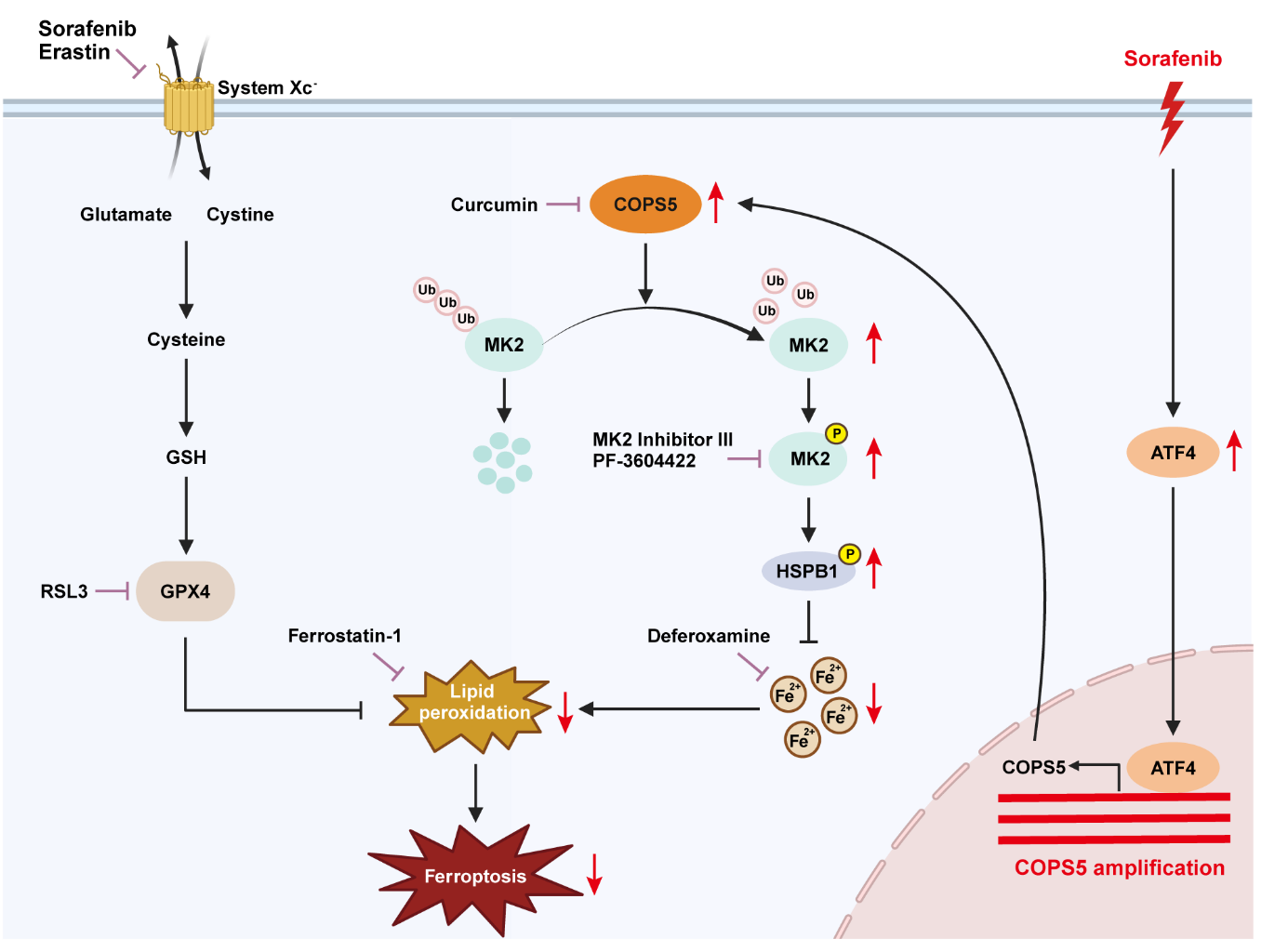


**Figure S14. Working model.** COPS5, which is overexpressed in HCC in an amplification- and ATF4-dependent manner, stabilizes MK2 through deubiquitination and, in turn, induces HSPB1 activation, protecting HCC cells from ferroptosis and thus promoting sorafenib resistance and tumor progression. This figure was created via BioRende.com.

# Supplementary references

[1]. M. Deng, L. Zhang, W. Zheng, J. Chen, N. Du, M. Li, W. Chen, Y. Huang, N. Zeng, Y. Song, Y. Chen. *J Exp Clin Cancer Res.* **2023**, 42, 9.

[2]. R. Pinyol, R. Montal, L. Bassaganyas, D. Sia, T. Takayama, G. Y. Chau, V. Mazzaferro, S. Roayaie, H. C. Lee, N. Kokudo, Z. Zhang, S. Torrecilla, A. Moeini, L. Rodriguez-Carunchio, E. Gane, C. Verslype, A. E. Croitoru, U. Cillo, M. de la Mata, L. Lupo, S. Strasser, J. W. Park, J. Camps, M. Sole, S. N. Thung, A. Villanueva, C. Pena, G. Meinhardt, J. Bruix, J. M. Llovet. *Gut.* **2019**, 68, 1065-1075.
